# Neuropilin-1 promotes mitochondrial structural repair and functional recovery in rats with cerebral ischemia

**DOI:** 10.1186/s12967-023-04125-3

**Published:** 2023-05-03

**Authors:** Ting Guo, Manli Chen, Ji Liu, Zengyu Wei, Jinjin Yuan, Wenwen Wu, Zhiyun Wu, Yongxing Lai, Zijun Zhao, Hongbin Chen, Nan Liu

**Affiliations:** 1 Department of Neurology, Fujian Medical University Union Hospital, Fuzhou, China; 2 Department of Rehabilitation, Fujian Medical University Union Hospital, Fuzhou, China; 3 Fujian Key Laboratory of Molecular Neurology, Fujian Medical University, Fuzhou, China; 4 Institute of Clinical Neurology, Fujian Medical University, Fuzhou, China; 5 Emergency Department, Fujian Medical University Union Hospital, Fuzhou, China

**Keywords:** Ischemia/reperfusion, NRP-1, Oxidative stress, Mitochondrial structure and function, Neuronal apoptosis

## Abstract

**Objectives:**

Available literature documents that ischemic stroke can disrupt the morphology and function of mitochondria and that the latter in other disease models can be preserved by neuropilin-1 (NRP-1) via oxidative stress suppression. However, whether NRP-1 can repair mitochondrial structure and promote functional recovery after cerebral ischemia is still unknown. This study tackled this very issue and explored the underlying mechanism.

**Methods:**

Adeno-associated viral (AAV)-NRP-1 was stereotaxically inoculated into the cortex and ipsilateral striatum posterior of adult male Sprague-Dawley (SD) rats before a 90-min transient middle cerebral artery occlusion (tMCAO) and subsequent reperfusion. Lentivirus (LV)-NRP-1 was transfected into rat primary cortical neuronal cultures before a 2-h oxygen-glucose deprivation and reoxygenation (OGD/R) injury to neurons. The expression and function of NRP-1 and its specific protective mechanism were investigated by Western Blot, immunofluorescence staining, flow cytometry, magnetic resonance imaging, transmission electron microscopy, etc. The binding was detected by molecular docking and molecular dynamics simulation.

**Results:**

Both in vitro and in vivo models of cerebral ischemia/reperfusion (I/R) injury presented a sharp increase in NRP-1 expression. The expression of AAV-NRP-1 markedly ameliorated the cerebral I/R-induced damage to the motor function and restored the mitochondrial morphology. The expression of LV-NRP-1 alleviated mitochondrial oxidative stress and bioenergetic deficits. AAV-NRP-1 and LV-NRP-1 treatments increased the wingless integration (Wnt)-associated signals and β-catenin nuclear localization. The protective effects of NRP-1 were reversed by the administration of XAV-939.

**Conclusions:**

NRP-1 can produce neuroprotective effects against I/R injury to the brain by activating the Wnt/β-catenin signaling pathway and promoting mitochondrial structural repair and functional recovery, which may serve as a promising candidate target in treating ischemic stroke.

## Introduction

Stroke has been ranked as a primary cause of disability and death in the elderly populace worldwide [1] and in the early phases of ischemic stroke, severe cell death and mitochondrial dysfunction may result in long-term neurological impairment [2]. As a countermeasure, vascular recanalization is currently the most efficacious treatment for acute ischemic stroke. However, its efficacy can be sufficiently compromised by the narrow window phase and subsequent ischemia and reperfusion (I/R) injury to the penumbra [3]. Therefore, it is of great significance finding an effective target to offset the I/R-induced neuronal damage.

Mitochondria, a powerhouse of the cell, are rich in a large number of enzymes, which not only participate in cellular oxidative phosphorylation and ATP synthesis, but also regulate intracellular calcium ions and reactive oxygen species to maintain cellular homeostasis. Studies have documented the involvement of mitochondria in the neuronal death following ischemic stroke [4]. In early ischemic stroke, mitochondrial structural damage and dysfunction can eventually lead to long-term neurological deficits [5]. During the early stage of cerebral I/R injury, significantly-swollen mitochondria decrease adenosine triphosphate (ATP) and produce excessive reactive oxygen species (ROS) and oxidative stress, finally resulting in cell death [6]. Moreover, excess ROS can open the mitochondrial permeability transition pore (mPTP), which induces the loss of mitochondrial membrane potential (ΔΨm) and the release of pro-apoptotic factors from the mitochondria into the cytoplasm, including apoptosis inducing factor (AIF) and cytochrome c (Cyt c) [7]. The latter in turn activates downstream Caspase-3 to induce cell apoptosis [8]. In addition, mitochondrial respiratory chain dysfunction directly leads to mitochondrial respiratory dysfunction and decreases oxygen consumption rate (OCR) [2, 9]. Therefore, promoting the mitochondrial structural repair and functional recovery is the crucial for the amelioration of the neurological damage after cerebral ischemia.

As a multifunctional, transmembrane, non-tyrosine kinase surface glycoprotein, neuropilin-1 (NRP-1) performs important functions in neural and vascular systems [10, 11]. A previous study has showed that in patients with COVID-19, NRP-1 serum level is associated with brain endothelial dysfunction, oxidative stress and the risk of thrombosis [12]. Another research has found that NRP-1 can exert mitochondrial protection by inhibiting excessive mitochondrial oxidative stress to maintain endothelial cell homeostasis and that it is also involved in maintaining mitochondrial membrane potential to increase cell activity [13]. In addition, NRP-1 can inhibit mitochondrial apoptosis by increasing the expression of the anti-apoptotic protein Bcl-2 and suppressing the transfer of the pro-apoptotic protein Bax from the cytoplasm to the interior of mitochondria [14]. Altogether, these findings evidence a pivotal role of NRP-1 in modulating mitochondrial function. However, it remains obscure whether NRP-1 can promote mitochondrial structural repair and functional recovery after cerebral ischemia and the underlying molecular mechanisms remains unexplored.

NRP-1 has been documented to participate in the maintenance of the stem cell properties of gastric cancer cells by activating the Wnt/β-catenin signaling pathway [15]. Wnt is well documented in the maintenance of stem cells, maturation of neurons, morphogenesis of dendrites, adult tissue homeostasis, and axonal remodeling [16]. As an downstream molecule of Wnt, β-catenin activates an array of target genes that participate in the homeostasis and survival of neurons in the central nervous system [17]. Without Wnt, β-catenin can be recognized in the cytoplasm by the Axin, adenomatous polyposis coliprotein (APC) and glycogen synthase kinase-3β (GSK-3β), subsequently phosphorylated by GSK-3β at sites of Ser 33, Ser 37, and Thr 41, and finally degraded by ubiquitination. However, the presence of Wnt can phosphorylate and inactivate GSK-3β, increasing the expression of β-catenin, which, in turn, results in the aggregation of β-catenin in the cytoplasm and entry into the nucleus to regulate the transcription of related target genes such as C-myc, Cyclin D1 [18]. Available literature has evidenced the role of the Wnt/β-catenin signaling pathway in the development and maturation of nerve cells, synaptic remodeling, dendritic cell morphogenesis, maintenance of intracellular homeostasis, regulation of cell death and survival, etc. [19]. Other studies have found that the activation of Wnt/β-catenin pathway can restore the damaged blood-brain barrier function and increase the reduced level of tight junction protein in AD [20] and can improve the integrity of the blood brain barrier by increasing the levels of tight junction proteins (occludin, claudin-5 and ZO-1), thereby promoting neural function in mice after stroke [21]. Recent studies demonstrate that the activation of the Wnt/β-catenin signaling pathway can improve the neurological function in rats with Parkinson’s disease by inhibiting mitochondrial oxidative stress, increasing the mitochondrial membrane potential, and promoting mitochondrial biogenesis [22] and that inhibiting Wnt/β-catenin signaling pathway can induce mitochondrial dysfunction, which further exacerbates cytotoxicity [23, 24]. Furthermore, p-β-catenin (Ser 37) can reinforce Bax expression and oxygen-glucose deprivation (OGD)-induced neuronal apoptosis [25]. Taken together, these findings evidence an essential involvement of the Wnt/β-catenin signaling pathway in regulating mitochondrial function. However, it remains unraveled whether this very signaling pathway is implicated in the NRP-1-promoted mitochondrial structural repair and functional recovery, thus alleviating the symptoms of neurological deficit in rats after cerebral ischemia.

The current study explored in vivo and in vitro the neuroprotection of NRP-1 against I/R injury to the mitochondrial structure and function in rat models and the related mechanism. The middle cerebral artery occlusion (MCAO) model and OGD/R model were established to investigate NRP-1-conferred neuroprotection against I/R injury to the brain. We found that NRP-1 overexpression restored motor function and structure of mitochondrial, maintained the mitochondrial integrity, and inhibited mitochondrial bioenergetic deficits, neuronal apoptosis, and oxidative stress. These protective effects were reversed by XAV-939, a Wnt/β-catenin signaling pathway inhibitor. These results signify that NRP-1 may serve as a promising candidate target in treating ischemic stroke.

## Materials and methods

### Animals

Adult Sprague-Dawley (SD) rats (male, 12-16-week-old, 250–280 g) were obtained from Experimental Animal Center of Fujian Medical University and housed in standard filter-top cages on a 12-h light/12-h dark cycle, with free access to water and food. The protocols observed the guidelines of National Institute of Health (NIH Publications NO. 80-23, revised in 1996). The experiments were approved by the Institutional Animal Care and Use Committee (IACUC) of Fujian Medical University in Fujian, China.

### Middle cerebral artery occlusion/reperfusion (MCAO/R) and experimental groups

A focal ischemic stroke was established in male SD rats by transiently occluding the middle cerebral artery (MCA) as described [26]. Briefly, throughout the surgery, the animals were intraperitoneally anesthetized using sodium pentobarbital (40 mg/kg), with body temperature kept between 36 and 37 °C using a feedback-controlled heating system (Harvard Apparatus, QC, Canada). A midline neck incision was made to expose the right common and external carotid arteries for ligation. A microvascular clip was placed on the internal carotid artery and a nylon monofilament (Diameter: 0.35-0.37 mm, Beijing Cinontech Co. Ltd, Beijing, China) was inserted through the internal carotid artery until a mild resistance was felt (approximately 18–20 mm). The monofilament was left in place for 90 min and then withdrawn. In the Sham group, animals received an identical surgery without the inserted intraluminal filament. The groups included: for the early stage, Sham, tMCAO, tMCAO + AAV-empty and tMCAO + AAV-NRP-1; for the late stage, Sham, tMCAO, tMCAO + AAV-NRP-1 and tMCAO + AAV-NRP-1 +XAV-939.

### Intracranial injection of adeno-associated virus and inhibitor

Rat models of cerebral I/R injury were established at Day 21 after the injection of AAV-NRP-1 or AAV-empty (Hanbio, Shanghai, China) into the cortex and ipsilateral striatum posterior to it [27]. Briefly, a Hamilton microlitre syringe (10 µl) was applied to the following coordinate axis, starting from the bregma: mediolateral [−]2.5 mm, anterior-posterior 0.2 mm, to a depth of 2.5 mm (cortex) and 4.5 mm (striatum). AAV-NRP-1 and AAV-empty (1.04 × 10^10^gc, respectively) were administered at a rate of 0.2 µl/min. After the injection, the syringe was left in place for at least 15 min to ascertain full virus diffusion and to prevent backflow of the virus. XAV-939 (inhibitor of Wnt/β-catenin signaling, 40 mg/kg) (Selleck Chemicals, TX, USA, Cat# S1180) was intraperitoneally injected 2 days before MCAO and on the day of the surgery before MCAO, respectively [28].

### Real-time quantitative PCR (qRT-PCR)

After reperfusion, TRIzol reagent (Takara, Tokyo, Japan, Cat# 9108) was adopted to extract total RNA from the peri-ischemic cortex tissues and the cultured neurons of rats. Subsequently, the total RNA was reverse transcribed to synthesize cDNA (Yeasen, Shanghai, China, Cat# 11141ES60). qRT-PCR was conducted using Applied Biosystems 7500 Real-time PCR System with SYBR Green Master Mix (Yeasen, Shanghai, China, Cat# 11202ES08). β-actin was treated as the internal control. The primers for RT-PCR included NRP-1: forward, 5’-AAGGAAACCTCGGTGGGATT-3’; reverse, 5’-GGTGCTCCCTGTTTCATCTATT-3’; β-actin: forward, 5’-TGAACCCTAAGGCCAACCGT-3’; reverse, 5′- GTACGACCAGAGGCATACAGG-3’. Relative NRP-1 mRNA expression was calculated by the 2^−ΔΔCT^ method and normalized to the mRNA levels of β-actin.

### Evaluation of neurological function

Neurological deficits were monitored before tMCAO, 12 h, 1 d, 3 d, and 7 d after MCAO/R induction. The sensorimotor performance of different groups was assessed with modified neurological severity scores (mNSS) that closely depict the correlation of the deficits with the overall severity of the histological injury [26]. Neurological deficits were assessed using a score scale of 0–18, with a higher score indicating a severer brain injury.

The rotarod test was mainly used to evaluate the dynamic balance of experimental animals with a rat rotarod 47,700 (Ugo Basile, Milan, Italy). The adaptive training of the test was started 3 days before tMCAO. The parameters were set to accelerate instrument from 4r/min to 40r/min within 300 s. The test was recorded 3 times a day, at an interval of 15 min in between. The duration of the animals’ staying on the rotating rod was measured. The means of the test data was used for statistical analysis.

The grip strength test was conducted to evaluate the neuromuscular function of the animals and the adaptive training was started 3 days before tMCAO. Briefly, the rats grasped with two forelimbs the grip plate of the grip strength tester (YLS-13 A, Yiyan Technology Development Co Ltd, Shan Dong, China). The tester pulled the rat backwards by the tail with the right hand and recorded the maximum pulling force that caused the rat to slip off the plate. The test was triplicated for each rat and the grip strength was presented as grams.

The cylinder test was designed to evaluate motor dysfunction [26]. Briefly, the rat was placed in a transparent plexiglass tube (20 cm in diameter of and 40 cm in height). When in the tube, the rat stood erect with one or both of its forelimbs placed on the wall of the tube to support the body. The rats were observed for 3 min and the number of the initial forepaw (left/right/both) placed on the wall of the tube was recorded. The rate of undamaged forelimb contact (right) was computed as follows: (right-left)/(left + right + both)×100%. The experiment was repeated three times for each rat.

### Magnetic resonance imaging (MRI) analysis

MRI (7-T, Bruker Medizintechnik, Germany) was used to visualize the infarct volume for all groups at Day 3 after ischemia-reperfusion. In short, rats were anesthetized with 1–3% isoflurane in a 1:4 oxygen/air mixture and then maintained with 100 µg/mL Dexmedetomidine Hydrochloride (0.15 µL/300 g b.w.). The T2-weighted imaging was performed by a fast spinecho (TubroRARE) sequence with the parameters set as follows: FOV = 35 × 35 mm^2^, TR/TE = 5200/32 ms, slice thickness = 0.56 mm, slices = 48, matrix = 256 × 256, total scan time = 11 min 5s 600ms. Respiration and heart rate were continuously monitored with an MRI-compatible physiological monitoring system (SurgiVet V3395TPR, Smiths medical, USA). The infarct volume (millimeters ^3^) was obtained by multiplying the summed infarct areas by the slice thickness (0.56 mm). The corrected infarct volume was computed with the following formula: corrected infarct volume = total contralateral half brain volume − (total ipsilateral half brain volume – total direct infarct volume). The quantification of the scanned images was performed with ImageJ software.

### Primary culture of cortical neurons

The primary cortical neurons were isolated and cultured as reported [29]. Briefly, 16-18-day-old embryonic rats were removed from the pregnant SD rats under anesthesia and their brains were quickly taken out. Tissues were dissociated by papain digestion and seeded on poly-L-lysine (100 µg/ml) plates in a neurobasal medium (Cat# 21103-049; Gibco, NY, USA) containing 2% B27 (Cat# 17504-044; Gibco, NY, USA), 0.5mM L-glutamine (Cat# 35050-061; Gibco, NY, USA) and 50 U/ml penicillin/streptomycin (Cat# 15140-122; Gibco, NY, USA). The neurons were cultured in a 5% CO2 chamber at 37 °C. The medium was firstly changed after 4 h and half-refreshed every other day before subsequent experiments.

### Oxygen and glucose deprivation/reoxygenation (OGD/R), lentivirus transfection and inhibitor

The OGD model was established with neurons cultured for 7–10 days, which mimicked the ischemia-like condition. In brief, the cell culture medium was refreshed with glucose-free DMEM (Cat# 11,966–025; Gibco, NY, USA), and cells were next transferred to a hypoxia environment (95% N_2_ and 5% CO_2_) for 2 h to induce injury. After OGD, cells were transferred to a normal medium and placed in a normoxic 5% CO_2_ incubator for 24 h. The negative control was not subjected to the OGD treatment. Cell transfection and the dosage of LV-NRP-1 (MOI = 5) were conducted according to the manufacturer’s instruction (Hanbio, Shanghai, China). Three days after the transfection, the transfection efficiency was monitored. Lentivirus without GFP was used for flow cytometry analysis and immunofluorescence. GFP-containing lentivirus-transfected neurons were subjected to additional experimental analysis. XAV-939 (10 µM)[30] were added into the NRP-1 medium immediately after OGD.

### Cell viability assay

The cell toxicity was tested by an enhanced cell counting kit-8 (CCK-8, Beyotime, Shanghai, China, Cat# C0041), following the manufacturer’s instructions [31]. Briefly, cells were seeded at 1 × 10^4^ (100 µl/well) in 96-well plates for 7 days. After a series of treatments, the CCK-8 reagent (10 µl) was administered to each well, and the plates were returned to the incubator for 2 h. The absorbance was read at 450 nm with a microplate reader (SpectraMax®i3x, Molecular Devices, USA).

The cellular activity of lactate dehydrogenase (LDH) was evaluated by detecting the release of LDH from neurons using a LDH Cytotoxicity Assay Kit (Beyotime, Shanghai, China, Cat# C0016), according to the manufacturer’s instructions [32]. Briefly, cells were seeded in 96-well plates with a density of 1 × 10^4^ cells/well for 7 days. After a series of treatments, the supernatant (120 µl) was combined with 60 µl LDH detection buffer, incubated at room temperature (RT) for 30 min and read at 490 nm with the same microplate reader.

### ROS quantifications

Neuronal ROS was detected using Dihydroethidium (DHE) dye (Beyotime, Shanghai, China, Cat# S0063). Cells were placed in six-well plates (3 × 10^5^ per well), incubated with 3 µM DHE at 37 °C for 30 min, and washed with PBS. Finally, cells were stained with DHE and imaged under a LeicaDMI8 microscope at ×20. ImageJ was used for processing and quantifying the DHE fluorescence intensity in images.

### Isolation of mitochondria, nuclear and cytosolic proteins

To isolate mitochondria from fresh peri-ischemic brain cortex, a Tissue Mitochondria Isolation kit (Beyotime, Shanghai, China, Cat# C3606) was used. Briefly, after the cortical tissue in ice-cold mitochondrial separation reagent A buffer was homogenized with 1 mM phenylmethanesulfonyl fluoride (PMSF) (Beyotime, Shanghai, China, Cat# ST506), the homogenate was centrifuged at 1000 g at 4 °C for 5 min. The collected supernatant was subject to further centrifugation of 11, 000 g at 4 °C for 10 min. For the protein analysis of mitochondria, the pellet containing isolated mitochondria was resuspended in mitochondrial lysate with 1 mM PMSF.

Following the manufacturer’s instructions, nuclear and cytosolic proteins were extracted with a Nuclear and Cytoplasmic Protein Extraction Kit (Cat# P0027; Beyotime, Shanghai, China). Cortical tissues were lysed in 200 µl of lysis buffer [cytoplasmic protein extraction reagent A (CPER A), CPER B, 1 mM PMSF] before centrifugation of 1500 *g* for 5 min at 4 °C. The supernatant was collected as partial cytoplasm. The pellet and cells were lysed in 200 µl of lysis buffer (CPER A, 1 mM PMSF). After placed on ice for 15 min, the pellet and cells received 10 µl of CPER B and centrifuged at 16, 000 g for 5 min at 4 °C, with the supernatant collected as cytoplasm. The pellet was further suspended with 50 µl of nuclear protein extraction reagent containing 1 mM PMSF and received a centrifugation of 16, 000 g for 10 min at 4 °C to collect the nucleoprotein-containing supernatant fraction.

### Western Blot

The peri-infarct cortex tissue or cultured primary neurons was homogenized with RIPA lysis buffer in the presence of 1% protease and phosphatase inhibitor cocktail and 1 mM PMSF. The samples received a centrifugation of 12, 000 r for 20 min at 4 ℃ to collect the supernatant. The concentration of protein was measured with a BCA Protein Assay Kit (Cat# 23227; Thermo Fisher Scientific, MA, USA). An amount of 20–60 µg of protein was then loaded onto an 8–12% SDS-polyacrylamide gel and separated by electrophoresis. The separated protein was transported from the gel to polyvin ylidene difluoride (PVDF) membranes (Millipore, MA, USA, Cat# ISEQ00010) and blocked using 5% bovine serum albumin (BSA, Meilunbio, MB4219) diluted with Tris-buffered saline containing 0.05% Tween 20 (TBST) at RT for 2 h. Subsequently, the membrane was incubated with various primary antibodies at 4 ℃ overnight. The primary antibodies included: rabbit anti-Neuropilin 1 (1:1000, Cat# ab81321; Abcam), rabbit anti-Bcl-2 (1:1000, Cat# A11313; ABclonal), rabbit anti-Bax (1:1000, Cat# 2772; Cell Signaling Technology), rabbit anti-caspase-3 (1:1000, Cat# 14220; Cell Signaling Technology), rabbit anti-Cyt c (1:1000, Cat# GB11080; Servicebio), rabbit anti-AIF (1:1000, Cat# A19536; ABclonal), rabbit anti-Wnt 3α (1:1000, Cat# A0642; ABclonal), rabbit anti-APC (1:1000, Cat# A17912; ABclonal), mouse anti-phospho-GSK3β-S9 (1:1000, Cat# AP1258; ABclonal), rabbit anti-Axin-1 (1:1000, Cat# A16019; ABclonal), rabbit anti-Wnt 1 (1:1000, Cat# A2475; ABclonal), rabbit anti-Cyclin D1 (1:1000, Cat# A19038; ABclonal), rabbit anti-c-Myc (1:1000, Cat# ab32072; Abcam), rabbit anti-β-Catenin (1:1000, Cat# 8480; Cell Signaling Technology), rabbit anti-β-actin (1:5000, Cat# YT0099; Immunoway), rabbit anti-Histone H3 (1:1000, Cat# 17562; ABclonal), mouse anti-Tomm20 (1:1000, Cat# ab56783; Abcam), rabbit anti-GSK3β (1:1000, Cat# 11731; ABclonal), rabbit anti-Phospho-beta catenin (Ser37) (1:1000, Cat# AF3266; Affinity Biosciences). After 3 washes with TBST, HRP-conjugated goat antibodies to rabbit (1:20000, Cat# AP132P; Millipore) or mouse (1:10000, Cat# AP124P; Millipore) IgG was applied at RT for 60 min. Finally, protein bands were visualized with the FluorChem M system (ProteinSimple, San Jose, CA, USA). The expression of protein was quantified with ImageJ software (Marlyand, USA).

### Immunofluorescence staining

Immunofluorescence staining was conducted as described previously [33]. Briefly, the slices were incubated with 22.5 mg/ml glycine in TBS for 30 min, and with blocking solution (10% donkey serum diluted with TBS with 3% BSA and 0.3% Triton X-100) at RT for 1 h. Then the slices were incubated with primary antibodies that were diluted in 2.5% donkey serum with TBS containing 1% BSA and 0.1% Triton X-100 at 4℃ overnight. The primary antibodies were used as follows: rabbit anti-Cyt c (1:100, Cat# GB11080; Servicebio), rabbit anti-Neuropilin 1 (1:100, Cat# PA5-95832; Thermo Fisher Scientific), rabbit anti-β-catenin (1:100, Cat# 8480; Cell Signaling Technology), mouse anti-NeuN (1:100, Cat# ab104224; Abcam). The slices were washed three times with TBST (TBS containing 0.2% Triton X-100). Cultured neurons were fixed in 4% paraformaldehyde-PBS (pH 7.4) for 15 min. Cells were permeabilised with 0.2% Triton X*-*100 for 15 min and then blocked with a blocking buffer (Cat#37581; Thermo Fisher Scientific, MA, USA) at RT for 1 h. Primary antibodies were diluted in PBS at 4℃ overnight, including rabbit anti-Neuropilin 1 (1:100, Cat# PA5-95832; Thermo Fisher Scientific), chicken anti-beta III Tubulin (1:1000, Cat# ab41489; Abcam), rabbit anti-Phospho-beta catenin (Ser37) (1:100, Cat# AF3266; Affinity Biosciences). After 3 washes with TBST or PBST (PBS containing 0.2% Triton X-100), the slices or cultured neurons underwent an incubation at RT for 2 h with the secondary antibodies: Donkey anti-Rabbit conjugated with Alexa Fluor 488 (1:1000, Cat# A21206; Thermo Fisher Scientific), Donkey anti-Mouse conjugated with Alexa Fluor 594 (1:500, Cat# A21203; Thermo Fisher Scientific), and Goat anti-Chicken conjugated with Alexa Fluor 594 (1:500, Cat# ab150172; Abcam). After 3 washes with TBST or PBST, brain slices or cultured neurons were stained with the nuclear stain 6-diamin-2-phenylindole-dihydrochloride (DAPI) (Cat# G1012; Servicebio) at RT for 10 min. After 3 washes with TBST or PBST, the images of slides were visualized under a confocal microscope (LSM 750, Zeiss, Gottingen, Germany).

### Transmission electron microscopy (TEM)

The mitochondrial ultrastructure and morphology of cortex tissues in SD rats were observed by TEM. After anaesthetization, rats were perfused with 0.1 M PBS (pH 7.4) and then with precooled PBS containing 1.5% paraformaldehyde and 3% glutaraldehyde. The cortex tissues (1 mm^3^) were fixed in a PBS mixture containing 1.5% ferrocyanide and then incubated in 1% osmium tetroxide for 2 h. After washing in 0.1 M PBS, tissues were dehydrated with a graded series of ethanol and acetone, and embedded in Epon and sliced for TEM. Ultrathin slices (90 nm) were prepared and stained with uranyl acetate and lead citrate. The mitochondrial ultrastructure was observed with a Tecnai G2 TEM (FEI company, OR, USA).

### Flow cytometry

Flow cytometry was performed to analyze cell apoptosis with an Annexin V-FITC Apoptosis Detection Kit (Cat# C1062L; Beyotime, Shanghai, China). Briefly, primary cortical neurons cultured in 25 cm^2^ flasks (3 × 10^6^ cells/flask) were obtained and then divided into 2 EP tubes (1.5 mL). The pellet in each tube was suspended in 195 µl of 1× annexin V-FITC binding buffer and 5 µl annexin V-FITC and incubated at RT for 10 min. After a centrifugation of 1000 g for 5 min, the pellet was resuspended in 10 µl of propidium iodide (PI) working solution and 190 µl of binding buffer. The samples were analyzed with a BD Accuri C6 Plus flow cytometer (BD Biosciences, CA, USA) and the data were processed with FlowJo 10.8.1 (BD Biosciences, CA, USA). The apoptotic percentage of neurons was indicated as the percentage of early apoptotic neurons + the percentage of late apoptotic neurons.

### TdT-mediated dUTP nick end labelling (TUNEL) staining

TUNEL staining was performed according to the manufacturer’s instructions (Cat# 11684817910; Roche Applied science, Penzberg, Germany). After a fixation with formalin, the slides were embedded in paraffin and sectioned. After deparaffinization, slides were subjected to enzyme reaction with the TUNEL reaction mixture before rinsing. The TUNEL reaction was performed using the freshly-prepared 3, 3-diaminobenzidine (DAB) solution and counter-staining was performed with hematoxylin. Apoptotic cells were observed by fluorescence microscopy (Olympus Corporation, Tokyo, Japan) with 20 × objective.

### Mitochondrial membrane potential measurement (ΔΨm)

The changes in the mitochondrial membrane potential (ΔΨm) were detected with a JC-1 kit (Beyotime, Shanghai, China, Cat# C2003S). In brief, primary cortical neurons cultured in 25 cm^2^ flasks (3 × 10^6^ cells/flask were acquired and subject to a centrifugation of 1, 000 rpm for 5 min. The pellet was incubated with 2 mL working solution in the dark at 37 °C for 20 min before another centrifugation of 1, 000 rpm for 5 min. After the pellet was washed with buffer solution, samples were detected with a BD Accuri C6 Plus flow cytometer (BD Biosciences, CA, USA). The data were processed with FlowJo 10.8.1 (BD Biosciences, CA, USA), with the results reported as the ratio of the red/green fluorescence.

### Lipid peroxidation and antioxidant measurements

Malondialdehyde (MDA) was measured with a Lipid Peroxidation MDA Assay Kit (Cat# S0131S; Beyotime, Shanghai, China). Superoxide Dismutase (SOD) activity was detected with a SOD Assay Kit (Cat# S0103; Beyotime, Shanghai, China). Glutathione (GSH)/Glutathione disulfide (GSSG) ratio was measured with a GSH and GSSG Assay Kit (Cat# S0053; Beyotime, Shanghai, China). Glutathione peroxidase activity (GSH-PX) was detected with a GSH-PX Assay Kit (Nanjing Jiancheng Bioengineering Institute, Nanjing, China, Cat# A005).

### ATP content

The intracellular ATP contents in neurons were detected with an Enhanced ATP Assay kit (Cat# S0027; Beyotime, Shanghai, China). Following the manufacturer’s instructions, pre-cooled lysis buffer (200 ml/well) was administered to the 6-well plate; the lysate was scraped with a cell scraper before a centrifugation of 12, 000 g at 4℃ for 5 min; and the supernatants were harvested. The ATP and protein concentrations were measured with a microplate reader (SpectraMax®i3x, Molecular Devices, USA) and the ATP content was normalized by protein concentration.

### Mitochondrial oxygen consumption rate (OCR)

The mitochondrial respiration in intact cells was analyzed with a Seahorse XFe24 Extracellular Flux Analyzer (Seahorse Bioscience, Copenhagen, Denmark). In brief, primary cortical neurons were placed into 24-well XFe24 culture plates (8 × 10^4^ cells/well). After 7 days, each well of the plate received Seahorse XF Calibrant Solution (Seahorse Biosciences, Copenhagen, Denmark). The sensor cartridge was soaked in Calibrant Solution at 37 °C overnight in a non-CO_2_ incubator and received the compounds existing in the Seahorse XF Cell Mito Stress Test Kit (Seahorse Bioscience, Copenhagen, Denmark, Cat# 103015-100) one hour before the measurement according to the protocol. The sensor cartridge was then placed into the Seahorse XFe24 Flux Analyzer for automated calibration and subsequently replaced by the XFe24 culture plates for bioenergetics analysis.

### Molecular dynamics (MD) trajectories analysis and molecular docking

Briefly, TIP3P water model was selected to add the composite system, consisting of the sodium ion (Na^+^) equilibrium system and a water box. The long-range electrostatic interaction was analyzed by particle mesh Ewald (PME) with elastic simulation calculated respectively by Verlet and cg scheme, at a cut-off value of 1.2 nm. The value of 10Å was used for non-bonded interactions. Energy minimization at a maximum of 50,000 steps was performed by the steepest descent method. Both the van der Waals radius cutoff distance and Coulomb force cutoff distance were set at 1.4 nm. The entire system was equilibrated using canonical (NVT) and the isothermal—isobaric (NPT) ensembles. The MD simulations were carried out for NRP-1, Wnt1, β-catenin, GSK-3β, Axin, APC for 100 ns. During the MD simulation, hydrogen atom-involved bonds were constrained using the *LINCS* algorithm, with the integration time step set at 2 fs. The temperature was maintained at 300 K with the V-rescale thermostat and the pressure was semi-isotropically kept at 1 bar with a Berendsen barostat. NVT and NPT equilibrations were respectively carried out at 30 ps under 300 K.

During the 100 ns simulation, the trajectory by MD simulation was obtained to investigate the motion behavior. MD was performed with the Gromacs 2019.6 software suite [34], using the Amber 14SB as the force field for proteins. Throughout the 100 ns MD simulation, analyzed were the radius of gyration (Rg), root mean square deviation (RMSD), hydrogen bond (HB) perturbations, and solvent accessible surface area (SASA).

The models of NRP-1, Wnt1, β-catenin, GSK-3β, Axin, APC were acquired from the Uniport Protein Data Bank [35]. The structures of NRP-1, β-catenin, Axin, Wnt1, GSK-3β were downloaded from AlphaFold2 [36]; APC was generated by Swiss-model [37]. The docking results were processed with HDock (https://pubmed.ncbi.nlm.nih.gov/28521030/) and visualized with Pymol and analyzed with LigPlot+. The binding free energies for every complex were calculate with the equations below:

$$\begin{aligned} \Delta G_{{bind}} & = \,\Delta {\text{G}}_{{{\text{complex}}}} - (\Delta {\text{G}}_{{{\text{receptor}}}} + \Delta {\text{G}}_{{{\text{ligand}}}} ) \\ & = \Delta {\text{E}}_{{{\text{internal}}}} + \Delta {\text{E}}_{{{\text{VDW}}}} + \Delta {\text{E}}_{{{\text{elec}}}} + \Delta {\text{G}}_{{{\text{GB}}}} + \Delta {\text{G}}_{{{\text{SA}}}} \\ \end{aligned}$$

where ∆E_VDW_, ∆E_internal_, ∆E_elec_ refer to, respectively, van der Waals, internal energy, and electrostatic energy. The solvation energy can be divided into the non-polar solvation free energy (G_SA_) and the electrostatic solvation free energy (G_GB_). Here, the ∆G_GB_ was calculated by the GB model developed by Nguyen (igb = 8). The ∆G_SA_ was estimated from the SASA using the Gmx_MMPBSA algorithm: ∆G_SA_ = 0.0072 × ∆SASA [38].

### **Statistical analysis**

Graphic presentation and statistical analysis were performed with GraphPad Prism v8.4.3 (GraphPad Software, San Diego, CA). Data were presented as mean ± standard error mean (SEM). The Shapiro-Wilk normality test and Brown-Forsythe test were respectively conducted to evaluate the normal distribution of the data and the homogeneity of variance. For data with normality and equal variance, one-way ANOVA with Bonferroni’s post hoc test was employed. Two-way repeated-measures analysis of variance (ANOVA) with Bonferroni’s post hoc test was applied to analyze the treatment effect on the behavior scores (mNSS score, rotarod test, grip strength test and cylinder test). A *p* value of less than 0.05 was deemed as statistically different.

## Results

### Ischemia upregulates the level of NRP-1 in cortical neurons

**Fig. 1:**
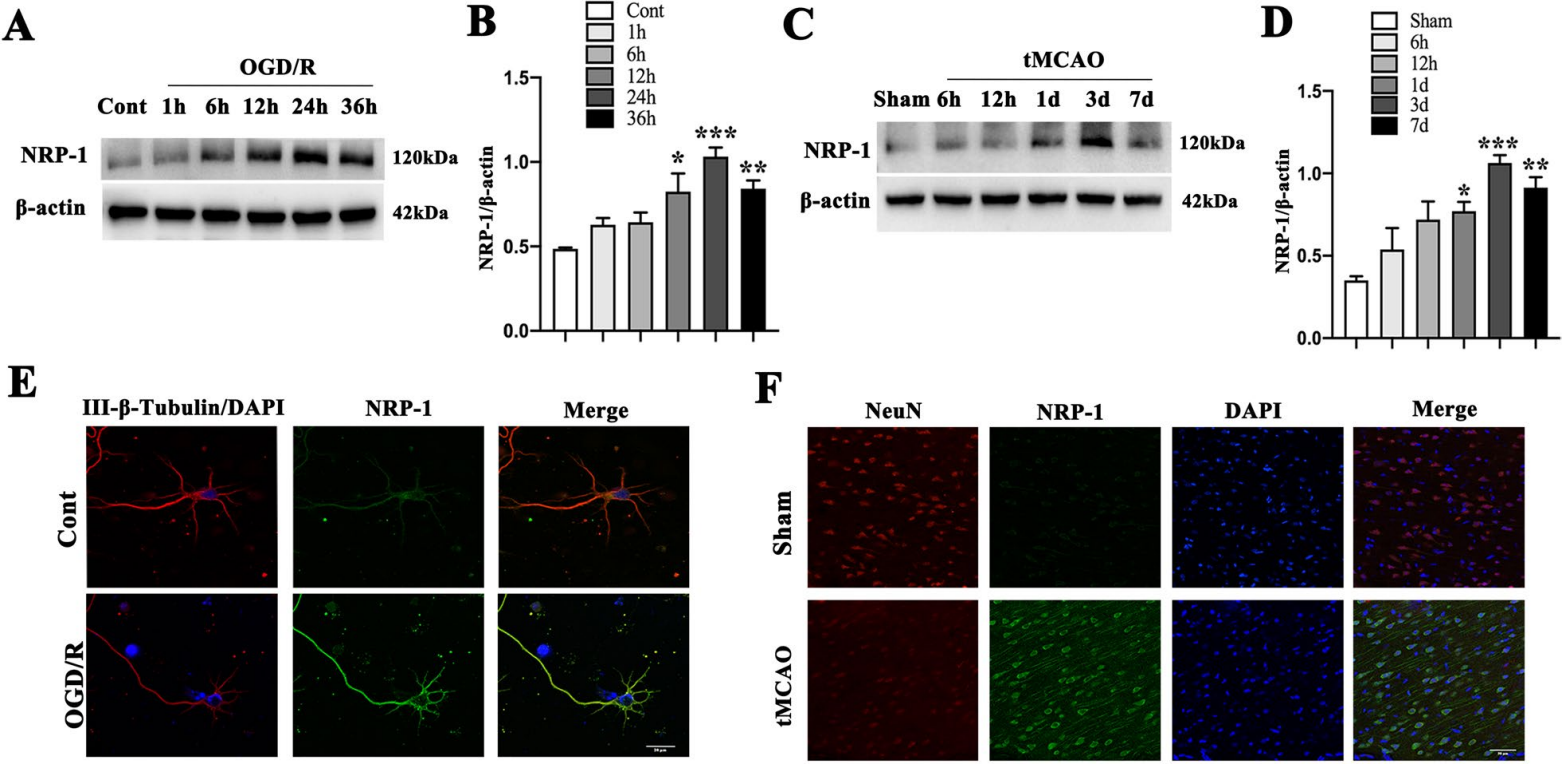
The increased level of NRP-1 in cultured primary neurons after OGD injury and in rats after tMCAO injury. **A** Representative immunoblots and **B** Western Blot analysis of NRP-1 in the cultured primary cortical neurons after OGD; n = 4. Data are expressed as mean ± SEM. *p < 0.05, **p < 0.01, ***p < 0.001, as compared with the Cont group by one-way analysis of ANOVA with Bonferroni’s post hoc test. **C** Representative immunoblots and **D** Western Blot analysis of NRP-1 in the penumbra region after tMCAO; n = 4. Data are expressed as mean ± SEM. *p < 0.05, **p < 0.01, ***p < 0.001, as compared with the Sham group by one-way analysis of ANOVA with Bonferroni’s post hoc test. **E** Localization of immunoreactive NRP-1 expression on neurons before and after OGD. The left column displays neuronal marker class III-β-Tubulin (red) and nucleus DAPI (blue). The middle column shows expression of NRP-1 (green). The right column shows the co-localization of NRP-1, class III-β-Tubulin and DAPI. Scale bar: 20 μm. **F** Localization of immunoreactive NRP-1 expression on neurons before and after tMCAO. The left column displays neuronal marker NeuN (red). The middle column shows expression of NRP-1(green). The right column displays nucleus marker DAPI (blue). The last column shows the co-localization of NRP-1, NeuN and DAPI. Scale bar: 50 μm. (Cont = Control)

Western Blot was performed to examine, at different time points, the ischemic responses of NRP-1 in the cultured primary rat cerebral cortical neurons that received OGD/reoxygenation (OGD/R). The analyses indicated that in comparison with that of the Cont group, the protein level of NRP-1 increased after OGD/R, with a significant upregulation at 24 h after OGD/R (Fig. 1A, B).

Western blot analyses were also conducted to evaluate the ischemic response of NRP-1 in the penumbra of the ischemic cerebral cortex of the 1.5 h-tMCAO rats. The results reported a markedly upregulated protein level of NRP-1 three days after tMCAO treatment when compared with that of the Sham group (Fig. 1C, D).

Immunofluorescence staining was performed to detect the expression of NRP-1 in neurons after OGD/R. As shown, at 24 h after OGD/R, immunoreactive NRP-1 expression was elevated in primary cortical neurons as in comparison with that of the Cont group (Fig. 1E). Fluorescent double immunostaining also confirmed the expression of NRP-1 in cortical neurons (specific with NeuN) in the penumbra of the ischemic cerebral cortex at 3 d after tMCAO as compared with that of the Sham group (Fig. 1F). These results together demonstrate that ischemia up-regulates NRP-1 level in cerebral cortical neurons in vitro and in vivo.

### Overexpression of NRP-1 ameliorates neuromotor function and reduces tMCAO-induced infarct volume

**Fig. 2:**
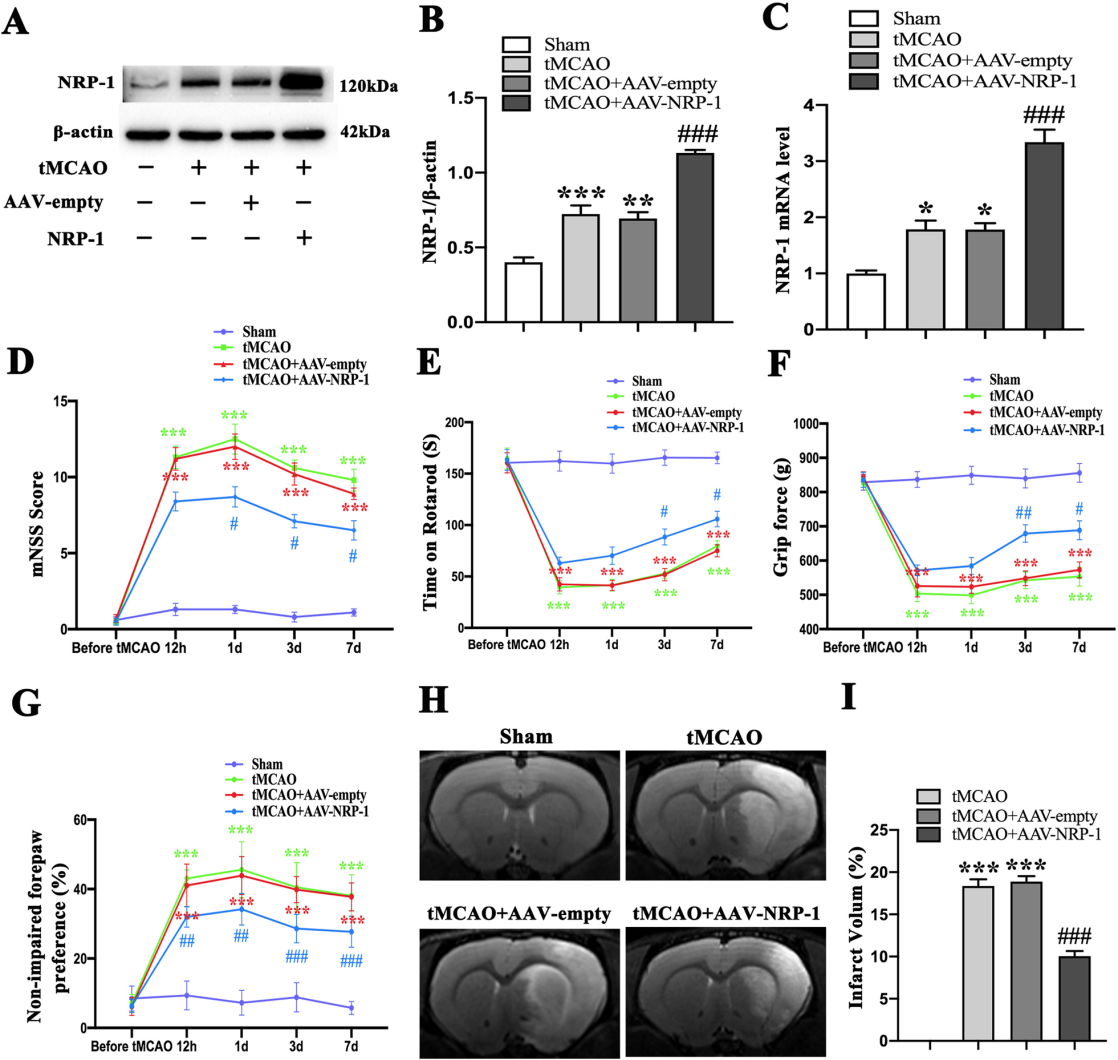
The improved functional recovery and reduced infarct volume by NRP-1 overexpression after tMCAO. **A** Representative immunoblots and **B** Western Blot analysis of NRP-1 in the penumbra region of different groups; n = 4. Data are expressed as mean ± SEM. and analyzed by one-way analysis of ANOVA with Bonferroni’s post hoc test. **p < 0.01, ***p < 0.001, as compared with the Sham group; ^**###**^*p* < 0.001, as compared with the tMCAO + AAV-empty group. **C** qPCR analysis of NRP-1 in the penumbra region of different groups. n = 5. Data are expressed as mean ± SEM. and analyzed by one-way analysis of ANOVA with Bonferroni’s post hoc test. *p < 0.05, as compared with the Sham group; ^**###**^*p* < 0.001, as compared with the tMCAO + AAV-empty group. **D** Modified neurological severity scores (mNSS), **E** rotarod test, **F** grip force test, **G** cylinder test were performed in different groups before tMCAO and 12 h, 1 d, 3 d and 7 d after tMCAO; n = 10. Data are expressed as mean ± SEM. and analyzed by repeated-measures analysis of variance (ANOVA) followed by Bonferroni’s multiple comparisons post-test. ***p < 0.001, as compared with the Sham group; ^**#**^*p* < 0.05, ^**##**^*p* < 0.01, ^**###**^*p* < 0.001, as compared with the tMCAO + AAV-empty group. **H** Representative T2 images of the brain at 3 d after ischemia and **I** the infarct volume in different experimental groups were plotted; n = 5. Data are expressed as mean ± SEM. and analyzed by one-way analysis of ANOVA with Bonferroni’s post hoc test. ***p < 0.001, as compared with the Sham group, ^**###**^*p* < 0.001, as compared with the tMCAO + AAV-empty group.

The neuroprotective effects of NRP-1 level on focal brain ischemic injury were investigated by injecting AAV-NRP-1 into rat cortex and striatum. Western Blot and qPCR analysis demonstrated that AAV-NRP-1 injection clearly increased the expression of NRP-1 (Fig. 2A–C). To further verify the functional recovery after AAV-NRP-1 administration in the tMCAO rat model, rats underwent mNSS score evaluation, rotarod test, cylinder test, and grip force test before tMCAO and at 12 h, 1d, 3d and 7d after tMCAO.

The mNSS score showed that AAV-NRP-1 treatment greatly attenuated neurobehavioral deficits at 3 d after tMCAO when in comparison with the tMCAO + AAV-empty group (Fig. 2D). Similarly, the tMCAO + AAV-NRP-1 group stayed markedly longer on the rod at 3d after tMCAO than the tMCAO + AAV-empty rats (Fig. 2E). Consistently, the grip force test reported improved grip strength in the tMCAO + AAV-NRP-1 group at 3 d after tMCAO when in comparison with that of tMCAO + AAV-empty rats (Fig. 2F). The cylinder test showed that compared with the tMCAO + AAV-empty group, the tMCAO + AAV-NRP-1 group reported a decrease in non-ipsilateral forepaw preference at 3 d after tMCAO (Fig. 2G). Moreover, the tMCAO + AAV-NRP-1 group showed markedly reduced infarct volume in the striatum and cortex at 3 d after tMCAO when in comparison with the tMCAO + AAV-empty group (Fig. 2H and I). Collectively, these findings evidence that the NRP-1 overexpression may provide neuroprotection against the neural damages induced by acute cerebral ischemia.

### Overexpression of NRP-1 inhibits neuronal apoptosis from cerebral ischemia/reperfusion injury

**Fig. 3:**
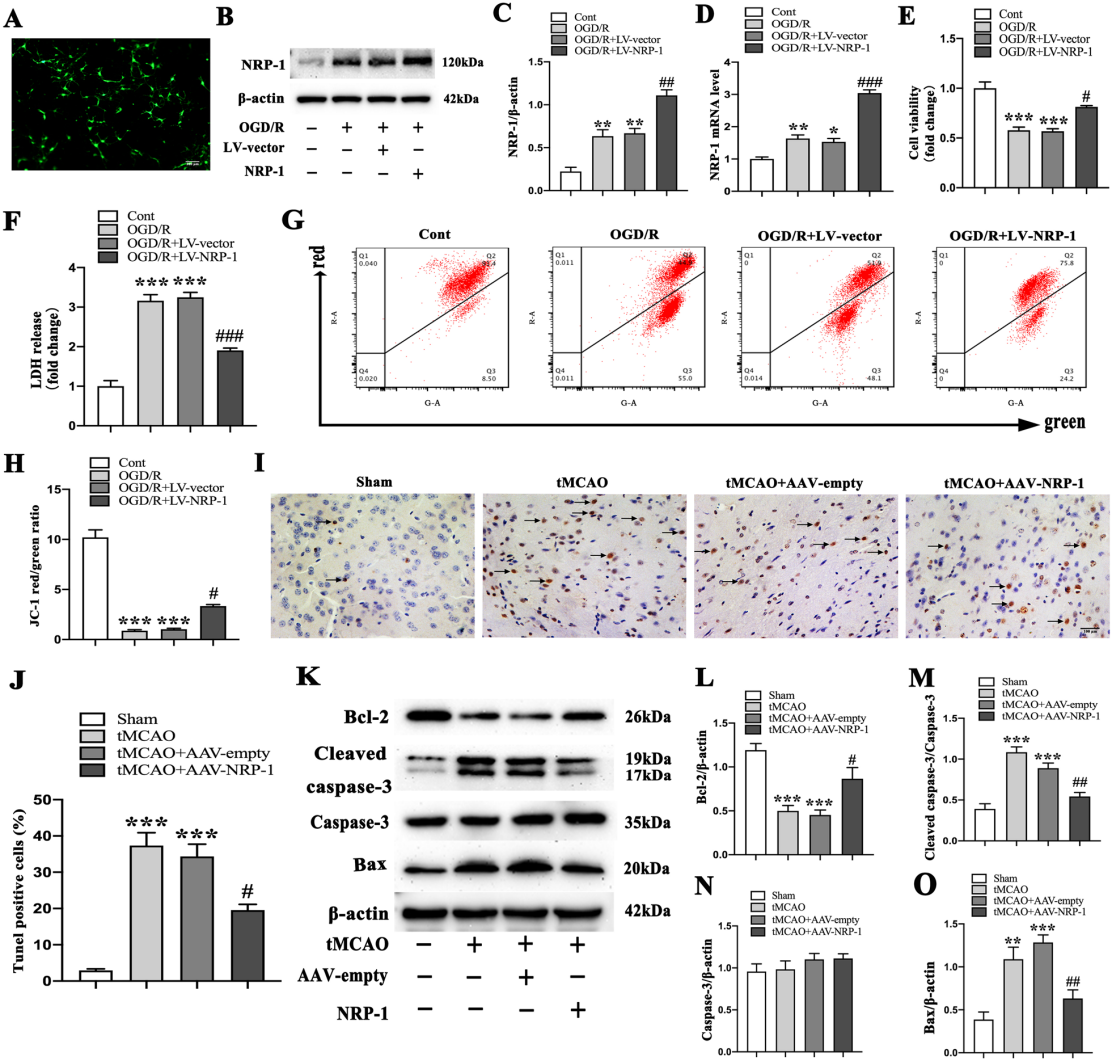
The inhibition of I/R-induced neuronal apoptosis by NRP-1 overexpression. **A** Representative immunofluorescence image exhibiting the overexpression of NRP-1 using GFP-containing lentivirus in cultured neurons. Scale bar: 200 μm. **B** Representative immunoblots and **C** Western Blot analysis of NRP-1 in cultured neurons of different groups; n = 4. Data are expressed as mean ± SEM. and analyzed by one-way analysis of ANOVA with Bonferroni’s post hoc test. **p < 0.01, as compared with the Cont group; ^**##**^*p* < 0.01, as compared with the OGD/R + LV-vector group. **D** qPCR analysis of NRP-1 in cultured neurons of different groups; n = 4. Data are expressed as mean ± SEM. and analyzed by one-way analysis of ANOVA with Bonferroni’s post hoc test. *p < 0.05, **p < 0.01, as compared with the Cont group; ^**###**^*p* < 0.001, as compared with the OGD/R + LV-vector group. **E** Cell viability was detected by CCK-8 assay; n = 3. Data are expressed as mean ± SEM. and analyzed by one-way analysis of ANOVA with Bonferroni’s post hoc test. ***p < 0.001, as compared with the Cont group; ^**#**^*p* < 0.05, as compared with the OGD/R + LV-vector group. **F** Cell cytotoxicity was determined by LDH release assay; n = 3. Data are expressed as mean ± SEM. and analyzed by one-way analysis of ANOVA with Bonferroni’s post hoc test. ***p < 0.001, as compared with the Cont group; ^**###**^*p* < 0.001, as compared with the OGD/R + LV-vector group. **G** Flow cytometry of JC-1 expression and **H** quantified results of red/green in cultured neurons of different groups; ***p < 0.001, as compared with the Cont group; ^**#**^*p* < 0.05, as compared with the OGD/R + LV-vector group. (Cont = Control). **I** Representative terminal deoxynucleotidyl transferase dUTP nick end labelling (TUNEL) images of different groups. Black arrows indicate TUNEL-positive cells. **J** the percentage of TUNEL-positive cells in the penumbra region of different groups; n = 3. Data are expressed as mean ± SEM. and analyzed by one-way analysis of ANOVA with Bonferroni’s post hoc test. ***p < 0.001, as compared with the Sham group; ^**#**^*p* < 0.05, as compared with the tMCAO + AAV-empty group. Scale bar: 100 μm. **K** Representative immunoblots and **L** Western Blot analysis of Bcl-2, **M** Cleaved caspase-3, **N** Caspase-3 and **O** Bax in the penumbra region of different groups; n = 4. Data are expressed as mean ± SEM. and analyzed by one-way analysis of ANOVA with Bonferroni’s post hoc test. **p < 0.01, ***p < 0.001, as compared with the Sham group; ^**#**^*p* < 0.05, ^**##**^*p* < 0.01, as compared with the tMCAO + AAV-empty group

To investigate the role of NRP-1 in the apoptosis of OGD-treated neurons, NRP-1 was overexpressed by LV interfering technique. The Western Blot and qPCR results showed that compared with the LV-vector group, the overexpression of NRP-1 successfully increased the levels of NRP-1 in primary cortical neurons at 24 h after OGD/R (Fig. 3A–D). Furthermore, the assays of LDH release and cell viability demonstrated that compared with the LV-vector group, the overexpression of NRP-1 in neurons cells decreased LDH release but improved cell viability at 24 h after OGD/R (Fig. 3E, F). The mitochondrial depolarization in living cells was measured with JC-1. The analyses reported that OGD/R resulted in an extensive depolarization of mitochondrial membrane potential (ΔΨm), as displayed by a marked decrease in the ratio of JC-1 aggregates (red fluorescence)/JC-1 monomer (green fluorescence) in the cultured neurons, while NRP-1 overexpression relieved this membrane depolarization (Fig. 3G, H).

Subsequently, the effect of NRP-1 on cellular apoptosis was explored in the tMCAO model. In the tMCAO + AAV-empty and tMCAO groups, the TUNEL assay showed a remarkable increase in TUNEL-positive cells in the penumbra of I/R-injured cortex when compared with the Sham group; whereas, in comparison with the tMCAO + AAV-empty group, the pre-treatment with AAV-NRP-1 decreased the number of TUNEL-positive cells at 3 d after tMCAO (Fig. 3I, J). Compared with the Sham group, the tMCAO group reported a decrease in Bcl-2 protein levels and an activation of Bax and Cleaved caspase-3. However, the tMCAO + AAV-NRP-1 group reported a lower expression of Bax and Cleaved caspase-3 and higher expression of Bcl-2 than the tMCAO + AAV-empty group. Meanwhile, no significant changes in pro-Caspase-3 levels were evident in each group (Fig. 3K–O). Altogether, these results indicate that NRP-1 can attenuate I/R-induced neuronal apoptosis and serve as a crucial inherent protection for cortical neurons during I/R.

### Overexpression of NRP-1 attenuates oxidative stress induced by cerebral ischemia/reperfusion

**Fig. 4:**
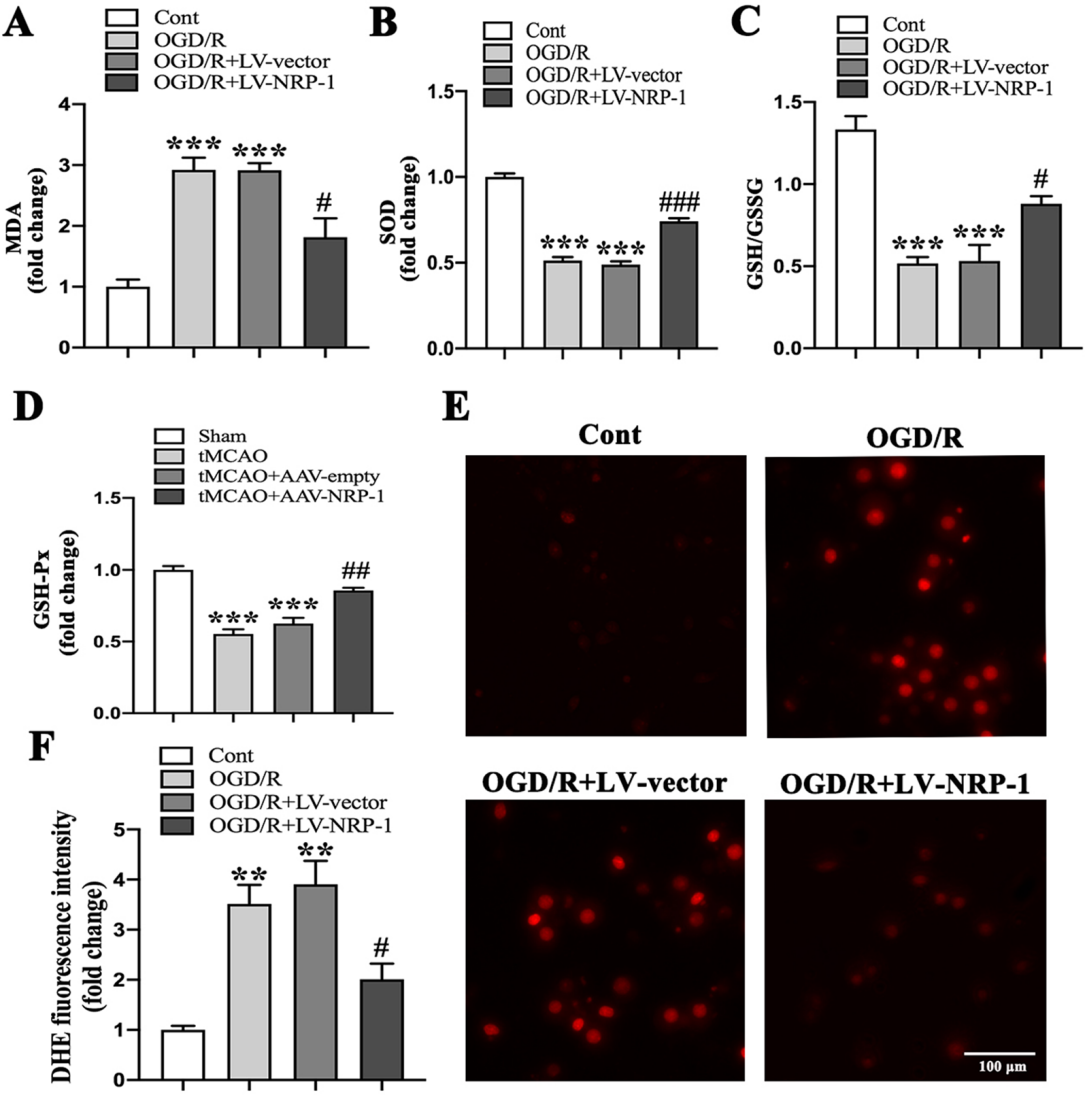
The attenuated oxidative stress by NRP-1 overexpression after ischemia/reperfusion injury. **A** Expression levels of MDA, **B** SOD, **C** GSH/GSSG in the neurons of different groups; n = 3. Data are expressed as mean ± SEM. and analyzed by one-way analysis of ANOVA with Bonferroni’s post hoc test. ***p < 0.001, as compared with the Cont group; ^**#**^*p* < 0.05, ^**###**^*p* < 0.001 as compared with the OGD/R + LV-vector group. **D** Expression levels of GSH-Px in the cortex of different groups; n = 3. Data are expressed as mean ± SEM. and analyzed by one-way analysis of ANOVA with Bonferroni’s post hoc test. ***p < 0.001, as compared with the Sham group; ^**##**^*p* < 0.01 as compared with the tMCAO + AAV-empty group. **E** Representative images of DHE staining. **F** Quantitative determination of fluorescence intensity in cultured neurons of different groups; n = 3. Data are expressed as mean ± SEM. and analyzed by one-way analysis of ANOVA with Bonferroni’s post hoc test. **p < 0.01, as compared with the Cont group; ^**#**^*p* < 0.05, as compared with the OGD/R + LV-vector group. Scale bar: 100 μm. (Cont = Control)

To further investigate the ischemia/reperfusion-induced oxidative damage and effects of NRP-1, a number of oxidative stress-related biochemical markers were measured. The results reported MDA increase, SOD decrease, and GSH/GSSG ratio decline in the OGD/R and OGD/R + LV-vector groups when in comparison with the Cont group; whereas, OGD/R + LV-NRP-1 treatment preserved the activities of SOD, GSH/GSSG and markedly suppressed the production of MDA, when in comparison with the OGD/R + LV-vector group (Fig. 4A–C). In addition, the level of GSH-Px decreased in the tMCAO and tMCAO + AAV-empty groups when in comparison with the Sham group; whereas, the AAV-NRP-1 treatment markedly promoted the production of GSH-Px, as in comparison with the tMCAO + AAV-empty group (Fig. 4D).

Since ROS is crucial in I/R injury, we also explored the effect of NRP-1 on resistance to oxidative stress. The results showed a dramatic increase of ROS production in the OGD/R + LV-vector and OGD/R groups, observed by the increased fluorescence of the ROS-sensitive probe DHE while the overexpression of NRP-1 significantly decreased DHE fluorescence intensity, indicating the decline of ROS level in the cultured primary neurons at 24 h after OGD/R (Fig. 4E, F). Taken together, the above results suggest that NRP-1 overexpression mitigates oxidative stress damage after cerebral I/R.

### NRP-1 overexpression promotes the mitochondrial structural recovery and attenuates mitochondrial bioenergetic deficits in cortical neurons after I/R injury

**Fig. 5:**
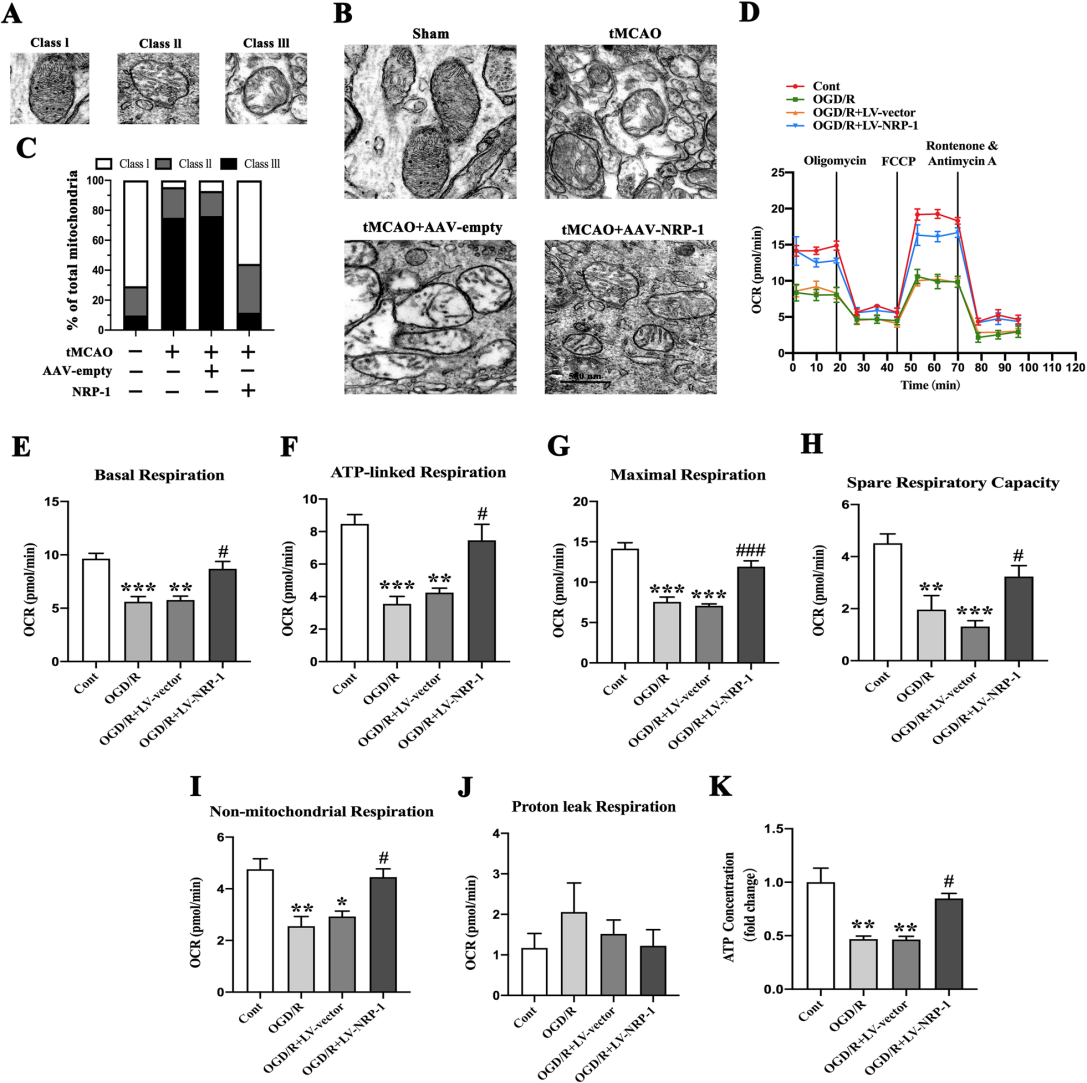
The enhanced mitochondrial structural recovery after tMCAO and increased oxygen consumption rates and ATP levels after OGD/R by NRP-1 overexpression. **A** Representative transmission electron microscopy (TEM) images of mitochondrial. **B** Representative TEM images in the penumbra region of different groups. **C** Quantification of mitochondrial morphological abnormalities. Mitochondria were classified into three types based on their morphologies: normal mitochondria (Class I), swollen and higher electron-dense mitochondria (Class II), and swollen and lower electron-dense mitochondria (Class III). A total of 40–50 mitochondria per experiment group were analyzed. n = 3. Scale bar: 500 nm. **D** Mean OCR of cortical neurons before and after the addition of oligomycin (1 µM), FCCP (1 µM), or rontenone (0.5 µM)/antimycin A (0.5 µM). Each data point represents an OCR measurement. **E** Quantitative results of basal respiration, **F** ATP-linked respiration, **G** maximal respiration, **H** spare respiratory capacity, **I** non-mitochondrial respiration. **J** proton leak respiration. n = 4; Data are expressed as mean ± SEM. and analyzed by one-way analysis of ANOVA with Bonferroni’s post hoc test. *p < 0.05, **p < 0.01, ***p < 0.001, as compared with the Cont group; ^**#**^*p* < 0.05, ^**###**^*p* < 0.001, as compared with the OGD/R + LV-vector group. **K** Quantified results of the levels of ATP in the cortical neurons of different experimental groups; n = 3. **p < 0.01, as compared with the Cont group; ^**#**^*p* < 0.05, as compared with the OGD + LV-vector group. (Cont = Control)

The alterations in mitochondrial morphology and biochemistry were further analyzed after ischemia/reperfusion. TEM observation revealed normal mitochondria (Class I), swollen and higher electron-dense mitochondria (Class II), and swollen and lower electron-dense mitochondria (Class III) (Fig. 5A). The brain cortex of the Sham group exhibited nearly 70% Class I mitochondria (Fig. 5B, C). However, in the tMCAO group, extensive damage was evident in the mitochondria, with approximately 20% Class II and 75% Class III mitochondria. Analogously, the mitochondrial ultrastructure in the tMCAO + AAV-empty group reported nearly 20% Class II, 75% Class III mitochondria. However, the overexpression of NRP-1 significantly reversed abnormal mitochondrial ultrastructure, reporting approximately 60% class I and 30% class II mitochondria. These results confirm that NRP-1 significantly restores the I/R-induced morphological abnormalities in mitochondria.

The OGD/R-induced impairment to the mitochondrial function was evaluated by analyzing the oxygen consumption rates (OCRs) of the cortical neurons with a Seahorse XFe24 analyzer (Fig. 5D). At 24 h after OGD/R treatment, basal respiration, maximal mitochondrial respiration, ATP-linked respiration, non-mitochondrial respiration, and the spare respiratory capacity were significantly down-regulated in OGD/R + LV-vector and OGD/R groups when in comparison with the Cont group; however, the NRP-1 overexpression partly increased OCR in OGD/R-injured cortical neurons (Fig. 5E–I). In addition, no differences in proton leak capacity were observed (Fig. 5J). Consistent with the decrease in OCR, the OGD/R and OGD/R + LV-vector groups, in comparison with the Cont group, reported a significant decrease in ATP content in cortical neurons. The overexpression of NRP-1 rescued the ATP content in OGD/R-injured cortical neurons when compared with the OGD/R + LV-vector group (Fig. 5K). Taken together, these results evidence that the overexpression of NRP-1 restores the mitochondrial morphology and promotes the recovery of mitochondrial functions after I/R injuries.

### Overexpression of NRP-1 preserves mitochondrial integrity in tMCAO rats

**Fig. 6:**
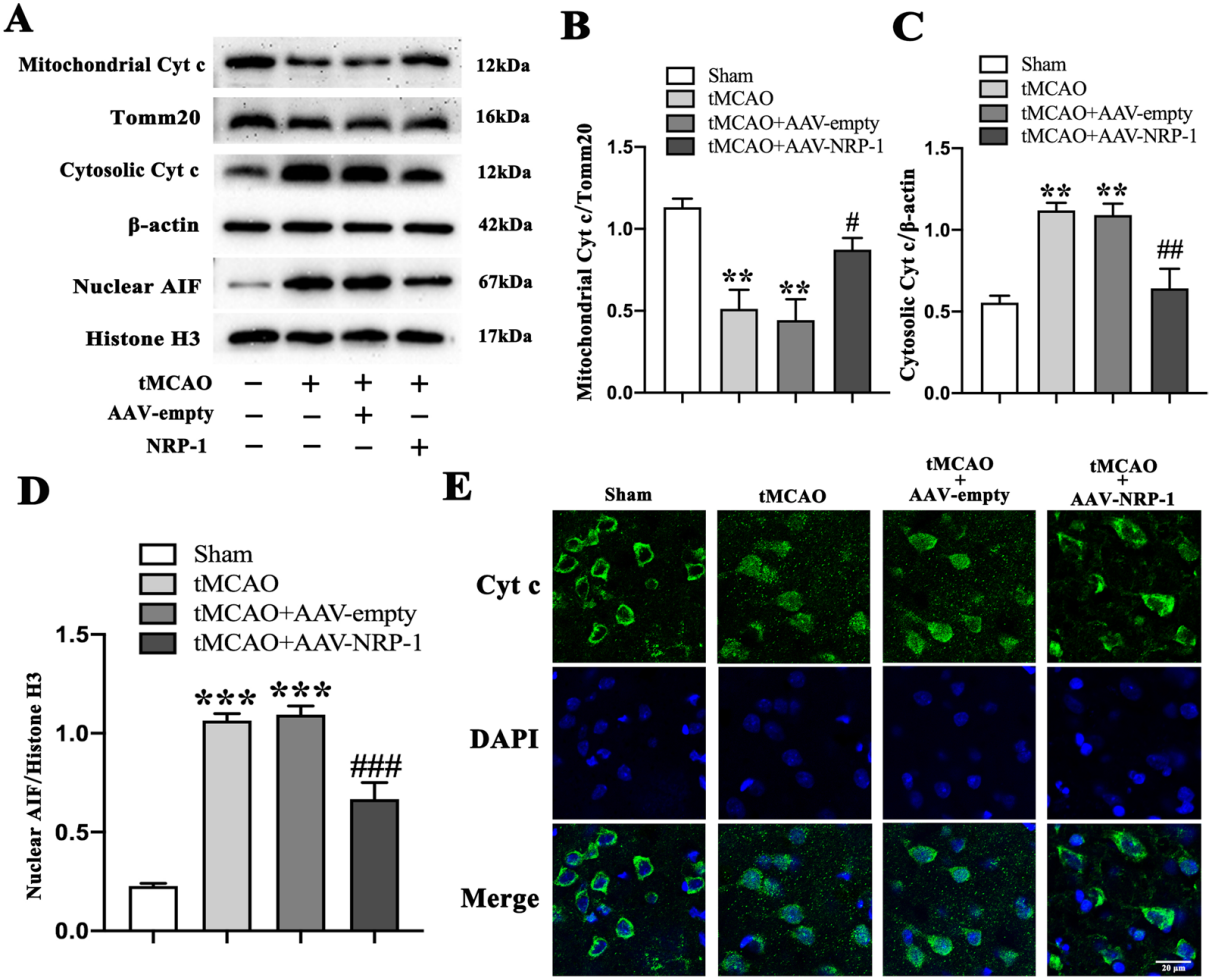
The preserved mitochondrial integrity by NRP-1 overexpression after tMCAO. **A** Representative immunoblots and **B** Western Blot analysis of mitochondrial Cyt c, **C** Cytosolic Cyt c, **D** Nuclear AIF in the penumbra region of different groups; n = 4. Data are expressed as mean ± SEM. and analyzed by one-way analysis of ANOVA with Bonferroni’s post hoc test. **p < 0.01, ***p < 0.001, as compared with the Sham group; ^**#**^*p* < 0.05, ^**##**^*p* < 0.01, ^**###**^*p* < 0.001, as compared with the tMCAO + AAV-empty group. **E** Representative immunofluorescence image of Cyt c in the cortex of different groups. Scale bar: 20 μm

To test the potential effect of NRP-1 on mitochondrial integrity after cerebral I/R injury, we examined Cyt c content in the cytosol and isolated mitochondria. In comparison with the Sham group, the tMCAO and tMCAO + AAV-empty groups reported a marked decrease in mitochondrial Cyt c levels but an increase in nuclear AIF and cytosolic Cyt c levels, indicating that ischemia/reperfusion induces a release of AIF and Cyt c from mitochondria. The overexpression of NRP-1 markedly diminished the AIF levels in the nuclei and Cyt c levels in the cytosol when in comparison with those of the tMCAO + AAV-empty group (Fig. 6A–D). Besides, Cyt c immunofluorescence showed the migration of Cyt c into the nucleus in the tMCAO and tMCAO + AAV-empty groups, while the NRP-1 overexpression attenuated the migration when in comparison with the tMCAO + AAV-empty group (Fig. 6E). Altogether, these findings suggest that NRP-1 overexpression plays a crucial role in the maintenance of mitochondrial integrity.

### NRP-1 overexpression-conferred mitochondrial protection is modulated through the Wnt/β-catenin signaling pathway

**Fig. 7:**
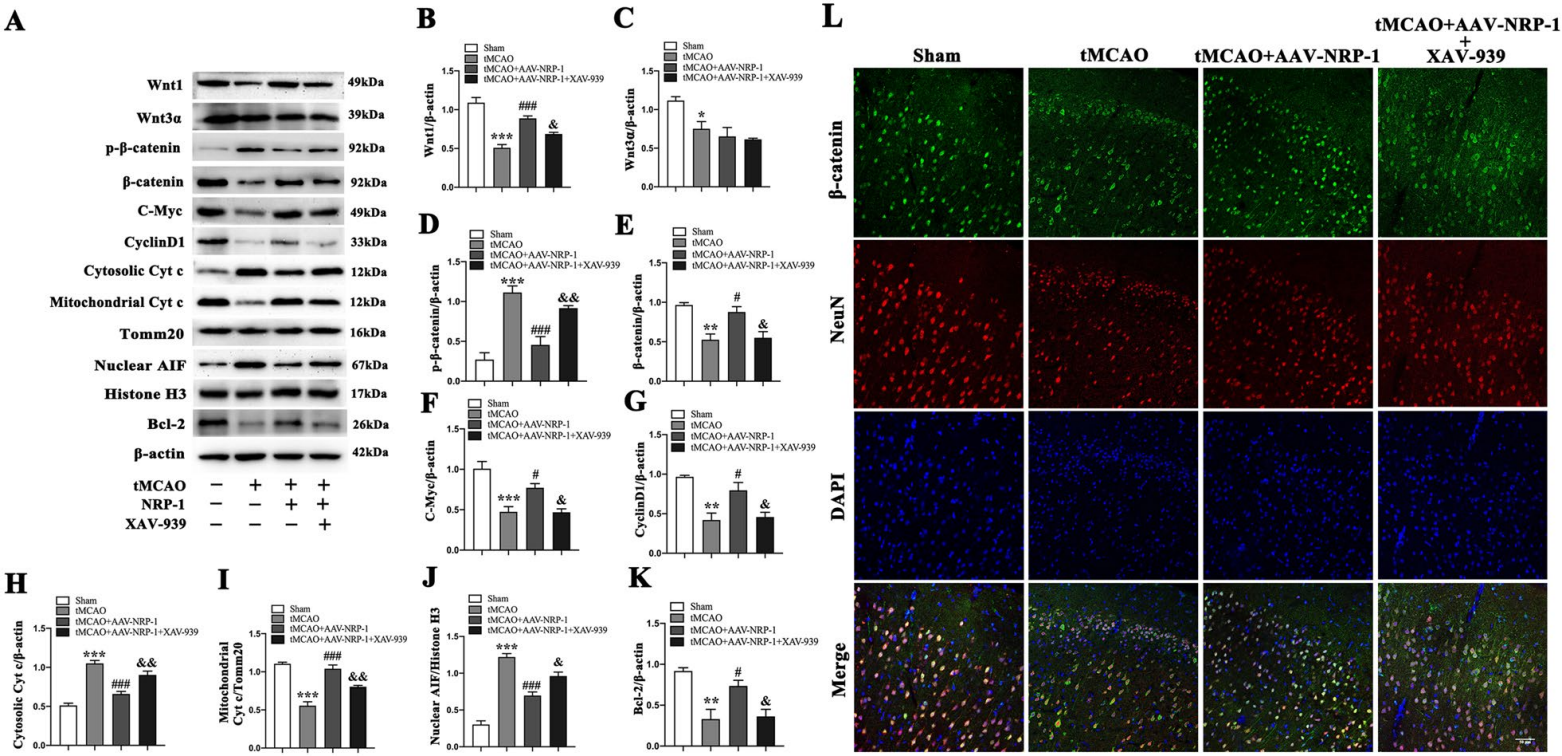
The effect of NRP-1 overexpression on protein expression in tMCAO lesions following administration of XAV-939. **A** Representative immunoblots and **B** Western Blot analysis of Wnt1, **C** Wnt3α, **D** p-β-catenin, **E** β-catenin, **F** C-Myc, **G** CyclinD1, **H** Cytosolic Cyt c, **I** mitochondrial Cyt c, **J** Nuclear AIF, **K** Bcl-2 in the penumbra region of different groups; n = 4. Data are expressed as mean ± SEM. and analyzed by one-way analysis of ANOVA with Bonferroni’s post hoc test. *p < 0.05, **p < 0.01, ***p < 0.001, as compared with the Sham group; ^**#**^*p* < 0.05, ^**###**^*p* < 0.001, as compared with the tMCAO group; ^**&**^*p* < 0.05, ^**&&**^*p* < 0.01, as compared with the tMCAO + AAV-NRP-1 group. (L) Representative immunofluorescence image of β-catenin in the penumbra region of different groups. Scale bar: 50 μm

To explore whether NRP-1 can activate the Wnt/β-catenin signaling pathway, the small molecule inhibitor XAV-939 (40 mg/kg; i.p) was employed to suppress the β-catenin-mediated transcription selectively. The expressions of Wnt1, Wnt3α, β-catenin, p-β-catenin (Ser 37), C-Myc, Cyclin D1, cytosolic Cyt c, mitochondrial Cyt c, Bcl-2, and nuclear AIF were detected by Western Blot. The analyses showed that compared with the Sham group, the tMCAO group reported a marked decrease in Wnt1, β-catenin, C-Myc, Cyclin D1, mitochondrial Cyt c and Bcl-2, but an increase in cytosolic Cyt c, nuclear AIF and p-β-catenin (Ser 37). Compared with the tMCAO group, the NRP-1 treatment increased the levels of β-catenin, Wnt1, Cyclin D1, C-Myc, mitochondrial Cyt c, and Bcl-2, but decreased the levels of cytosolic Cyt c, nuclear AIF, and p-β-catenin (Ser 37). Interestingly, XAV-939 reversed these changes. Except for the Sham group, all the other groups reported a sharp decrease in Wnt3α (Fig. 7A–K).

To further confirm the effects of NRP-1 overexpression on the Wnt/β-catenin signaling pathway in the neurons, the immunofluorescence labelling was performed to detect the distribution of β-catenin at 3 d after tMCAO. The results of the double immunofluorescence staining revealed a marked reduction of β-catenin nuclear localization in the tMCAO group when in comparison with the Sham group. However, NRP-1 overexpression promoted localization of β-catenin to the nucleus when in comparison with the tMCAO group, which served as a hallmark of canonical Wnt pathway activation. The nuclear translocation of β-catenin was reduced by XAV-939 (Fig. 7L). These findings demonstrate that the NRP-1 overexpression activates the Wnt/β-catenin signaling pathway, up-regulates β-catenin expression, and reduces p-β-catenin (Ser 37) vitality in cortical neurons after tMCAO, which is partly subject to XAV-939-induced neutralization.

### **The NRP-1 overexpression alleviates the OGD-induced apoptosis of primary cultured cortical neurons by the Wnt/β-catenin signaling pathway**

**Fig. 8:**
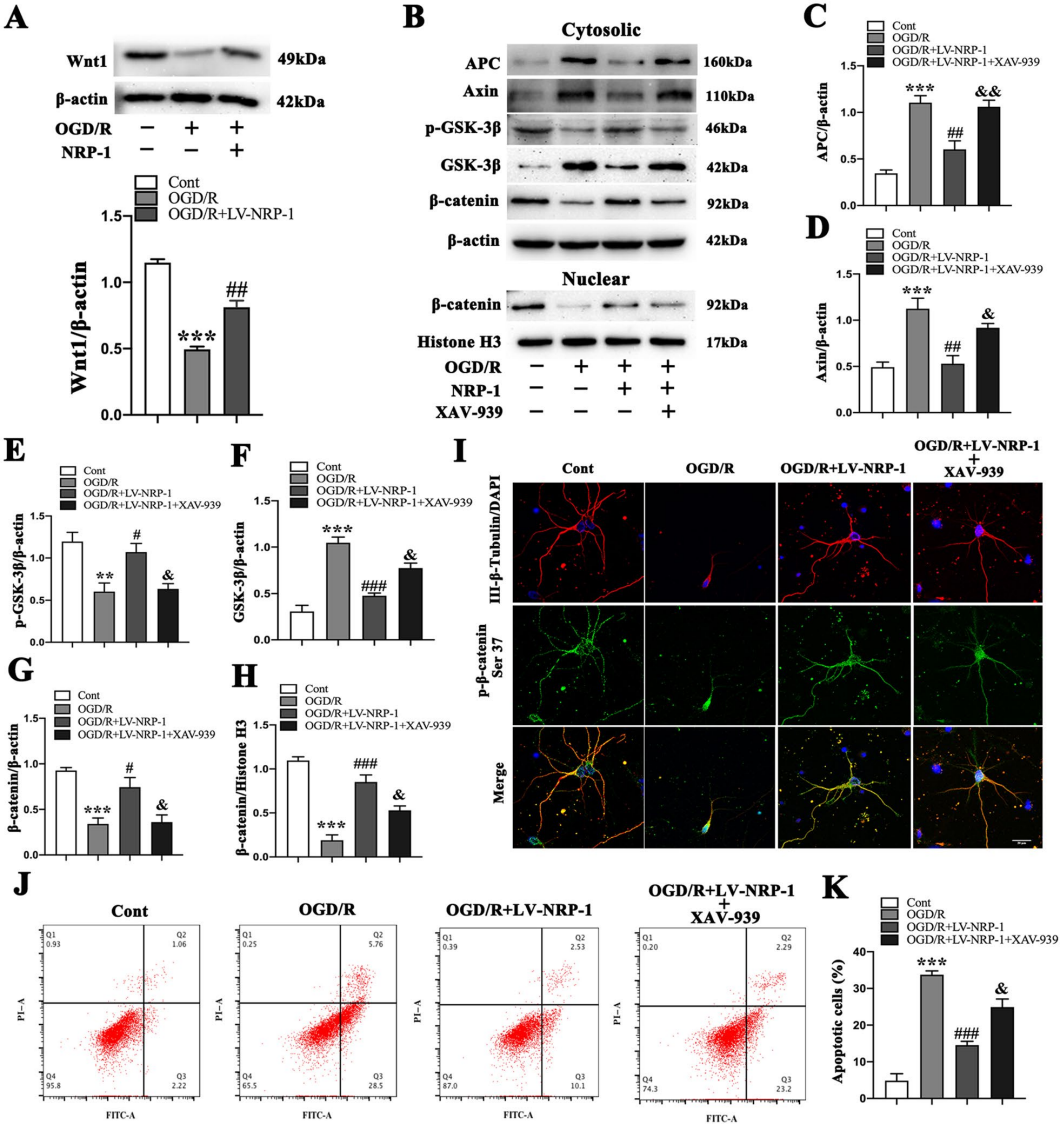
The attenuated OGD-induced apoptosis in cultured primary cortical neurons by NRP-1 overexpression via the Wnt/β-catenin signaling pathway. **A** Representative immunoblots and Western Blot analysis of Wnt1; n = 4. Data are expressed as mean ± SEM. and analyzed by one-way analysis of ANOVA with Bonferroni’s post hoc test. ***p < 0.001, as compared with the Cont group; ^**##**^*p* < 0.01, as compared with the OGD/R group. **B** Representative immunoblots and **C** Western Blot analysis of APC, **D** Axin, **E** p-GSK-3β, **F** GSK-3β, **G** β-catenin in the cytoplasm and **H** β-catenin in the nucleus of different groups; n = 4. Data are expressed as mean ± SEM. and analyzed by one-way analysis of ANOVA with Bonferroni’s post hoc test. **p < 0.01, ***p < 0.001, as compared with the Cont group; ^**#**^*p* < 0.05, ^**##**^*p* < 0.01, ^**###**^*p* < 0.001, as compared with the OGD/R group; ^**&**^*p* < 0.05, ^**&&**^*p* < 0.01, as compared with the OGD/R + LV-NRP-1 group. **I** Representative immunofluorescence image of p-β-catenin (Ser37) in cultured neurons of different groups. Scale bar: 20 μm. **J**–**K** The apoptosis of cultured neurons in different groups by flow cytometry; n = 3. Data are expressed as mean ± SEM. and analyzed by one-way analysis of ANOVA with Bonferroni’s post hoc test. ***p < 0.001, as compared with the Cont group; ^**###**^*p* < 0.001, as compared with the OGD/R group; ^**&**^*p* < 0.05, as compared with the OGD/R + LV-NRP-1 group. (Cont = Control)

To further clarify the mechanism underlying the NRP-1-induced activation of the Wnt/β-catenin signaling pathway, the specific pathway inhibitor XAV-939 was applied to neurons after OGD. We next detected Wnt1 protein by Western Blot. The analyses showed that OGD/R significantly decreased the expression of Wnt1 when compared with the Cont group and that the NRP-1 overexpression upregulated the expression of Wnt1 (Fig. 8A). We next detected APC, β-catenin, GSK-3β, Axin, and p-GSK-3β proteins in the pathway by Western Blot. The analyses showed that OGD/R significantly increased the expressions of APC, GSK-3β, and Axin but reduced the p-GSK-3β level in cytosol when compared with those of the Cont group. The NRP-1 overexpression-induced activation of the Wnt/β-catenin signaling pathway upregulated the expression of p-GSK-3β in cytosol but downregulated the expressions of APC, Axin, and GSK-3β in cytosol. However, the treatment with XAV-939 decreased the level of p-GSK-3β but increased that of APC, Axin, GSK-3β in cytosol (Fig. 8B–F).

Subsequently, we detected the level of β-catenin, a central molecular effector of canonical Wnt signaling pathway. Compared with the Cont group, the OGD/R group showed significantly-reduced β-catenin level in the cytosolic and nuclear fraction of neurons. Compared with the OGD/R group, the NRP-1 treatment upregulated the level of β-catenin in the cytosolic and nuclear fraction; compared with the OGD/R + LV-NRP-1 group, XAV-939 decreased the level of β-catenin in the cytosolic and nuclear fraction (Fig. 8G–H). The immunofluorescence labelling was also used to detect the distribution of p-β-catenin (Ser37) at 24 h after OGD. The results revealed that compared with the Cont group, the OGD/R group reported an increase in p-β-catenin (Ser37) nuclear localization, which was reduced by NRP-1 overexpression when in comparison with the OGD/R group but increased by XAV-939 when in comparison with the OGD/R + LV-NRP-1 group (Fig. 8I).

We further determined the number of apoptotic cells by flow cytometry. As indicated in Fig. 8J, K, the proportion of apoptotic cells rose sharply in the OGD/R group when in comparison with that of the Cont group and declined in the OGD/R + LV-NRP-1 group when in comparison with the OGD/R group. However, XAV-939 increased the apoptosis when in comparison with the OGD/R + LV-NRP-1 group. These findings indicate that NRP-1 overexpression activates the Wnt/β-catenin signaling pathway by disrupting the β-catenin degradation complex, which inhibits the cellular apoptosis following OGD/R injury.

### The simulations of molecular dynamics and calculations of binding free energy

**Fig. 9:**
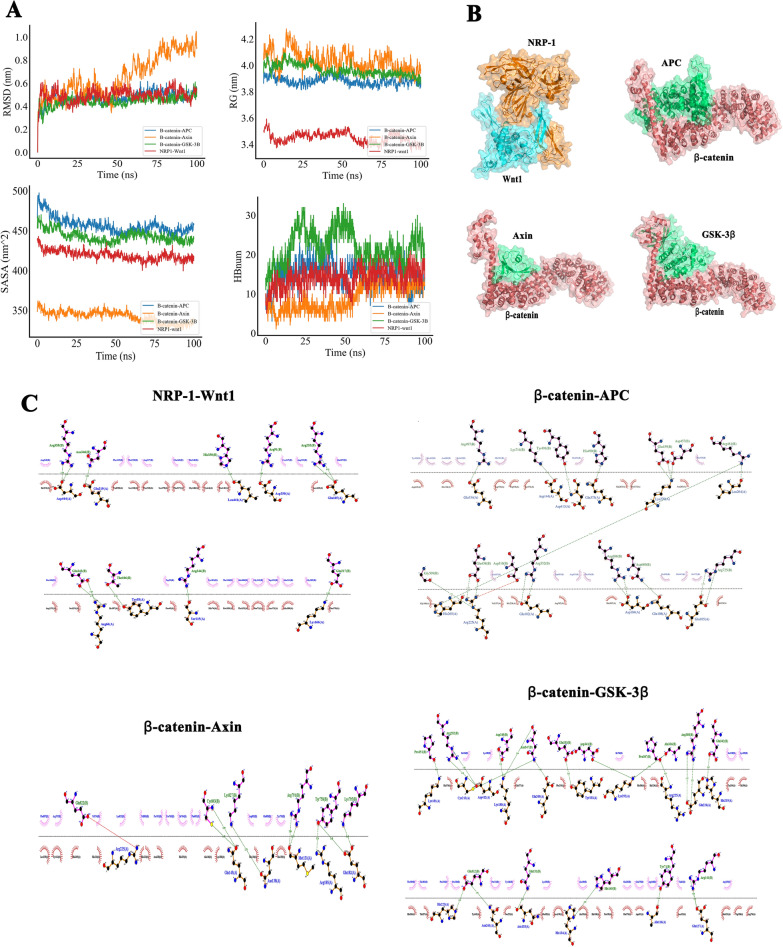
The analysis of Molecular Dynamics Simulation (MDS) results. **A** Root mean square deviation (RMSD), radius of gyration (Rg), solvent accessible surface area (SASA) and hydrogen bond (HB) profiles of the NRP-1 with Wnt1, the complexes of β-catenin with APC, Axin, and GSK-3β systems. **B** The binding mode of NRP-1 to Wnt1, β-catenin to APC, Axin and GSK-3β, respectively. **C** The specific views of the 2-D ligand interaction among NRP-1 with Wnt1, β-catenin with APC, Axin and GSK-3β, respectively. (B-catenin = β-catenin, GSK-3B = GSK-3β).

MD (100 ns) was performed to assess the underlying involvement of NRP-1 in mediating the Wnt/β-catenin signaling pathway. RMSD was adopted to observe the conformational changes of the system during the simulation process and to measure the stability of the system. β-catenin with APC, NRP-1 with Wnt1, and GSK-3β systems were stabilized in the initial simulation phase of 0–10 ns and maintained at 0.6 nm. Rg was employed as a proxy for the compactness of a structure. After initial brief fluctuations, all systems quickly entered a state of equilibrium. SASA was a parameter to measure the fraction of the protein surface interacting with the solvent molecules. The analyses revealed that the SASA of the protein decreased steadily in 0-100 ns, indicating a favorable binding of each system and a gradual protein fluctuation. From the HB change curve, the contact of the β-catenin with GSK-3β system ranged between 10 and 30 times, while that of the β-catenin with Axin system was 5–10 times (Fig. 9A). After MD simulations, the binding interactions of NRP-1with Wnt1 and β-catenin respectively with APC, Axin, and GSK-3β were examined (Fig. 9B). The results showed that the NRP-1 and Wnt1 formed an X-shaped contact with a large contact area and that β-catenin-APC, β-catenin-Axin, and β-catenin-GSK-3β systems displayed similar V-shaped contact patterns. The contact fingerprint was further depicted to indicate the contact force and properties (Fig. 9C).

**Fig. 10:**
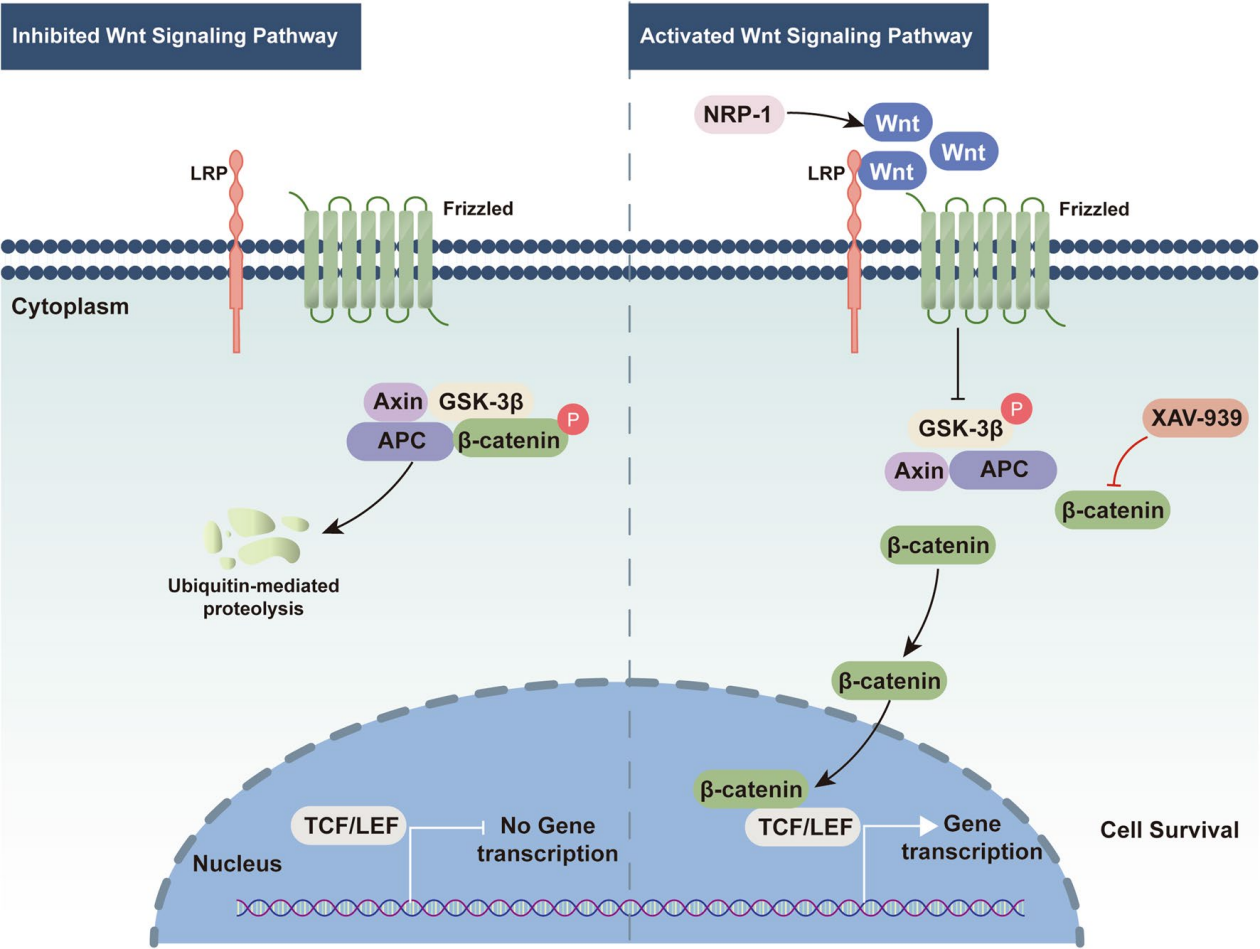
The schema of the protective mechanism of NRP-1 overexpression in I/R injury. Abbreviations: NRP-1, Neuropilin-1; Wnt, wingless integration; LRP, low-density lipoprotein receptor-related protein; GSK-3β, glycogen synthase kinase-3β; APC, adenomatous polyposis coli; Axin, axis inhibition protein

The difference in free energies between the two solvated proteins was calculated in the bound and unbound states, respectively, and the free energy binding of different solvated conformations of the same molecule was compared (Table 1). The results reported negative free energies between protein complexes, ranging from − 35.95 kcal/mol, − 51.98 kcal/mol, − 75.47 kcal/mol, to − 93.17 kcal/mol, respectively, in which the binding free energy of β-catenin-GSK-3β was the lowest, indicating the strongest affinity between GSK-3β and β-catenin. A comparison among β-catenin-Axin, β-catenin-APC, and β-catenin-GSK-3β revealed that the electrostatic energy of β-catenin and GSK-3β, and the van der Waals energy were the strongest (− 513.99 kcal/mol and − 217.75 kcal/mol, respectively), dominating the contribution to the binding free energy between the complexes when in comparison with that of APC and Axin. In the composition of the free energy of NRP-1-Wnt1, despite a strong electrostatic energy between the two (− 2053.06 kcal/mol), the polar solvation energy (2149.38 kcal/mol) decreased the total free energy. These interactions evidence the tight connections between NRP-1 and Wnt1 and between β-catenin and APC, Axin, GSK-3β, respectively. The overexpression of NRP-1 increases the expression of Wnt proteins. Wnt proteins are released to the extra-cellular space for the binding to Frizzled/LRP receptors, which induces the phosphorylation of GSK-3β and prevents the destruction complex (APC/ GSK-3β/Axin) from the degradation of β-catenin. Preserved β-catenin enters the nucleus and interacts with the TCF-LEF promoter complex, leading to the activation of related target genes and promoting cell survival. The XAV-939 prevents the degradation of Axin, allowing the accumulation of the degradation complex (APC/GSK-3β/Axin), which allows β-catenin degradation for cell death (Fig. 10).

**Table 1:** Complex free energy distribution (kcal/mol)

Group	VDWAALS	EEL	EGB	ESURF	GGAS	GSOLV	TOTAL
β-catenin-APC	− 174.50	− 364.55	526.35	− 23.25	− 539.05	503.10	− 35.95
β-catenin-Axin	− 97.50	− 203.69	263.65	− 14.44	− 301.19	249.21	− 51.98
β-catenin-GSK-3β	− 217.75	− 513.99	668.45	− 29.88	− 713.74	638.57	− 93.17
NRP1-Wnt1	− 151.35	− 2053.06	2149.38	− 20.44	− 2204.41	2128.94	− 75.47

VDWAALS: van der waals energy; EEL: electrostatic energy; EGB: polar solvation energy; ESURF: non-polar solvation energy; GGAS: total gas phase free energy; GSOLV: total solvation free energy; TOTAL: GSOLV + GGAS.

## **Discussion**

The present study demonstrated that NRP-1 was an important protective factor for the early I/R-induced injury to cerebral cortical neurons. It also showed that after I/R injury, the overexpression of NRP-1 significantly attenuated OGD/R-induced cytotoxicity in neurons and enhanced neurological functional recovery. In addition, the NRP-1 overexpression reduced neuronal apoptosis by downregulating the activation of caspase-3 and Bax and upregulating Bcl-2. Finally, the NRP-1 overexpression ameliorated mitochondrial dysfunction by suppressing mitochondrial bioenergetic deficits and oxidative stress, repairing mitochondrial morphology and ultrastructure, and maintaining mitochondrial integrity in the post-ischemic brain. This NRP-1 overexpression-conferred protection from cerebral I/R insult was accompanied by the up-regulation of Wnt and down-regulation of APC, GSK-3β, Axin, which inactivated the destruction complex, inhibited the phosphorylation of β-catenin at Ser37, and upregulated the β-catenin level in nucleus and cytoplasm. However, this protection was inhibited by β-catenin inhibitor XAV-939, All the findings evidence that after cerebral I/R injury, the NRP-1 overexpression activates the Wnt/β-catenin signaling pathway, signifying the neuronal-specific protective mechanism of the NRP-1/Wnt/β-catenin pathway in ischemic neurons.

NRP-1 has been documented in neurons, T-cells, dendritic cells and migrating cancer cells [39–41]. A previous study has reported an increased expression of endothelial NRP-1 in the ischemic cerebral hemispheres, which helped to prevent the cellular damage in cerebrovascular diseases [42]. Data suggest that NRP-1 in the endothelial cells is up-regulated and contributes to neovascularization in post-ischemia brain [43]. Similarly, a marked increase in NRP-1 was evident after I/R-induced cerebral injury in rats and OGD/R insult to cortical neurons. We found that ischemia-injured neurons were intensively receptive to NRP-1 and peaked at 24 h after OGD/R and 3 d after tMCAO. As NRP-1 has a protective effect on ischemic injury, we speculate that NRP-1 may be an endogenous protective factor. However, unlike our finding, a previous study reported that NRP-1 mRNA expression peaked at 6 h in cerebral cortex after permanent MCAO [44]. This difference may be due to different rat species and animal models. The current study employed AAV and LV to modulate the expression of NRP-1. We found that the transfection of AAV and LV-NRP-1 significantly upregulated NRP-1 levels when in respective comparison with AAV-empty and LV-vector transfection, which showed that the AAV-NRP-1 and LV-NRP-1 functioned well.

Neurological function and brain tissue are very sensitive to ischemia-reperfusion injury [45]. After cerebral ischemia/reperfusion, deteriorated motor coordination, weakened muscular strength, and asymmetrical forelimb use frequently occur due to the sensory deficits and contralateral limb motor dysfunction. In this study, we explored the effect of NRP-1 on neurological function in rats after I/R injury by assessing the rotarod performance, mNSS scores, grip force, and forelimb symmetry. We provide the first evidence that transfected NRP-1 promoted neurological recovery by improving locomotor coordination, asymmetry in forelimb use and muscle strength. In addition to enhanced neurological function, the infarct size was significantly reduced. The above results indicate that NRP-1 overexpression ameliorates the tMCAO-induced cortical damages and improves the behavioral deficits.

Apoptosis, a programmed cell death mechanism in neurons characterized by DNA fragmentation, is now recognized as an important factor in accelerating tissue injury and cell death after cerebral ischemia [46]. Mitochondrial damage is one of the important causes of apoptosis [47], in which ischemia-hypoxia induces mitochondrial stress, inducing the mitochondrial depolarization and eventual cell death [48]. Our findings revealed that NRP-1 promoted the survival of neurons, reduced the neurotoxicity of neurons, and increased mitochondrial membrane potential, which illustrates that the overexpression of NRP-1 exerts neuroprotective effects on neurons by inhibiting apoptosis. To further explore the anti-apoptotic effect of NRP-1, we detected the TUNEL-positive cells and protein levels of Bax, Bcl-2, Cleaved caspase-3, and Caspase-3 in tMCAO rats. A recent study evidences that TUNEL-positive cells are apoptotic in nature [49]. The Bcl-2 family and the cysteine proteases known as caspases play an important role in apoptosis, in which both Bax and Bcl-2 belong to the Bcl-2 family, with the former as an anti-apoptotic gene and the latter as an pro-apoptotic gene[50]. Caspase-3 is the caspase most directly associated with apoptosis[51]. Previous studies find that NRP-1 protects rheumatoid synoviocytes from apoptosis by mediating Bax translocation and Bcl-2 expression [14, 52]. This study found that NRP-1 reduced TUNEL-positive cells and the expression of active Caspase-3 and Bax, and upregulated the expression of Bcl-2 in the peri-ischemic region after tMCAO, which is consistent with previous findings that NRP-1 can protect rheumatoid synoviocytes from apoptosis by increasing the expression of Bcl-2 and suppressing the transfer of Bax from the cytoplasm to the interior of mitochondria [14]. The findings of this study reveal that NRP-1 overexpression prevents ischemia-induced neuronal apoptosis by suppressing the pro-apoptotic genes and increasing the anti-apoptotic gene (Bcl-2).

Oxidative stress is a typical pathological feature of cerebral I/R injury and has been strongly involved in the neuronal death [32]. The stress results from the imbalance between ROS and antioxidant systems [53, 54]. Excessive generation of ROS, MDA, and decreased activity of antioxidant enzymes such as SOD, GSH-Px and GSH have been observed during oxidative stress[55, 56]. As the key ROS metabolic biomarker, MDA is the product of unsaturated lipid degradation by ROS, whose level in the cells can reflect the severity of oxidative stress damage. However, antioxidant systems, GSH-Px, GSH and SOD can directly scavenge free radicals such as ROS. Recent studies indicate that NRP-1 suppresses oxidative stress by reducing mitochondrial ROS and promoting the production of superoxide SOD in endothelial cells [13]. Our results showed that NRP-1 decreased the I/R-induced excessive ROS and MDA production by improving the antioxidant system, such as increasing the activity of SOD, GSH-Px and the rate of GSH/GSSG, in line with the previous study that NRP-1 can inhibit excessive mitochondrial oxidative stress to maintain endothelial cell homeostasis [13]. Altogether, these findings indicate that NRP-1 protects neuronal cells against oxidative stress-induced impairment, which highlights an attractive and novel therapeutic strategy.

The disruption of the mitochondrial structure is evident in the neurons following cerebral ischemia [57]. When cells undergo apoptosis, mitochondrial swelling, decreased density, the shape of mitochondrial cristae, fusion of individual crista, and widening of crista junctions cause complete release of Cyt c and mitochondrial dysfunction [58]. As mitochondrial structure has been demonstrated to modulate the extent and efficiency of mitochondrial respiration and ATP production, the preservation of mitochondrial structure is essential for proper mitochondrial function[59, 60]. In the current study, TEM analysis revealed that NRP-1 overexpression alleviated mitochondrial swelling and increased the electronic density of mitochondria, the number of mitochondrial cristae, related bioenergetic parameters, and ATP synthesis, indicating mitochondrial structural repair, robust upregulation of mitochondrial bioenergetics, and improved mitochondrial activity after neuronal ischemia. Taken together, the administration of NRP-1 improves mitochondrial structure in the tMCAO model and mitochondrial bioenergetics in the OGD/R model.

I/R injury results in the loss of mitochondrial integrity and subsequent apoptosis [48]. Hypoxia or free radicals may induce an increased permeability of mitochondrial pores, releasing Cyt c and AIF from the mitochondria to the cytoplasm. The released Cyt c combines with Caspase-9 and Apaf-1 to form an “apoptotic body”, which in turn activates Caspase-3 and results in apoptosis [61]. AIF can be transferred from cytosol to nucleus, inducing DNA degradation and cell death [62]. The decreased release of AIF and Cyt c in the NRP-1-treated group indicates that NRP-1 can reduce the release of AIF and Cyt c by improving the mitochondrial membrane potential and membrane integrity. The above results evidence that NRP-1 may inhibit the release of AIF and Cyt c by improving the mitochondrial integrity, and protect neurons from I/R-induced injury.

The Wnt/β-catenin signaling pathway is crucial for the regulation of various cellular events, including preventing cell apoptosis [63, 64]. Recent studies have shown that the classical Wnt signal transduction can induce neuronal growth in adults after injury to the central nervous system, suggesting that the developmental role of Wnt can be replicated in adults by exogenous stimulation [65]. Recent research has reported enhanced antagonistic activity and decreased Wnt ligands in neurodegenerative and excitotoxic disorders [66, 67]. After stroke, the intranasal delivery of recombinant Wnt3α protein has been found to contribute to strong regeneration, neuroprotection, and improved functional outcomes [30]. Endogenous Wnt1 expression is significantly lost while Wnt1 overexpression maintains microglial integrity, activity, and proliferation during OGD exposure [68]. The results showed that the expression of Wnt1 and Wnt3α were decreased after cerebral ischemic injury, also consistent with previous studies that document the decrease of Wnt1 and Wnt3α expression in the penumbra region of the hemisphere after MCAO [28]. The results showed that overexpressed NRP-1 increased the expression of Wnt1, but not that of Wnt3a, which indicates that Wnt1 is involved in NRP-1-induced activation of Wnt/β-catenin signaling pathway.

Available studies report that when Wnt is upregulated, the Wnt/β-catenin signaling pathway is activated, inhibiting the phosphorylation of β-catenin [28]. The stabilized β-catenin is accumulated in the cytoplasm and translocated into the nucleus to regulate the expression of specific genes such as Cyclin D1, C-Myc [69]. As a key regulators of the cell cycle, Cyclin D1 mainly facilitates cell proliferation and correlates positively with the cell viability after I/R injury [70]. As an intranuclear oncogene, C-Myc encodes nuclear DNA binding proteins and engages in apoptosis, cell cycle progression, and cellular transformation [71]. It is also associated with stem cell activity under hypoxic conditions [69]. Previous studies indicate that cerebral I/R can decrease the protein expressions of Cyclin D1, β-catenin, and C‐Myc [72] and that binding of 14-3-3ζ to p-β-catenin Ser37 may reduce the degradation of β-catenin, thereby increasing total β-catenin to reduce cell death [73]. Other studies have shown that in OGD-treated neurons, the elevation of p-β-catenin Ser37 promotes cell death [25]. Consistent with these findings, we found that NRP-1 inhibited the phosphorylation of β-catenin Ser37 and promoted the expression of downstream genes (Cyclin D1 and C-Myc). Next, to confirm whether the Wnt/β-catenin signaling pathway is activated by NRP-1 overexpression, XAV-939, a specific antagonist of this signaling pathway, was used for subsequent testing. XAV-939 has been found to inhibit tankyrase, thereby preventing the degradation of Axin and allowing the accumulation of the degradation complex, which has been utilized to exacerbates the neuronal damage [30, 74]. Other studies demonstrate that XAV-939 negatively regulates the expression of Wnt/β-catenin signaling pathway in rats after ischemic stroke [30]. Consistent with previous studies, in our study, the i.p. injection of XAV-939 successfully inhibited the Wnt/β-catenin signaling pathway and reversed the NRP-1-conferred protection.

Next, we further verified the protective effect of NRP-1 on neuronal cells by activating the Wnt/β-catenin signaling pathway at the cellular level. Firstly, XAV-939 was used to probe into the mechanisms underlying the Wnt/β-catenin signaling pathway-related effects. Our findings indicated that the NRP-1 overexpression promoted the expression of Wnt1, signifying that NRP-1 protects neurons after OGD/R by activating the Wnt/β-catenin signaling pathway. Next, we analyzed the expression of the degradation complex to investigate the molecular mechanism in NRP-1-promoted neuronal viability after OGD injury. The initiation of the canonical Wnt pathway causes dissociation of the β-catenin degradation complex (Axin/APC/GSK-3β). Then, the phosphorylation of β-catenin by GSK-3β at Thr41, Ser33, and Ser37 resulted in the aggregation of β-catenin in the cytoplasm and eventually in the nucleus [75, 76]. A recent study documents that after cerebral ischemia, the increased β-catenin degradation complex inhibits the activity of the Wnt/β-catenin signaling pathway [72]. The current study found that NRP-1 promoted the nuclear translocation of β-catenin by inhibiting the degradation complex, which were partly attenuated by XAV-939 after OGD/R. In addition, representative micrographs of immunostaining showed that NRP-1 decreased the endogenous translocation of p-β-catenin Ser37 into the nucleus of cultured neurons at 24 h after OGD, which was reversed by XAV-939, indicating that NRP-1 activates the Wnt/β-catenin signaling pathway by increasing Wnt expression and inhibiting the degradation complex. Furthermore, in the current study, we found that XAV-939 reversed the protective effect of NRP-1 against neuronal apoptosis. Recent studies demonstrate that the activation of the Wnt/β-catenin signaling pathway can improve the neurological function by inhibiting mitochondrial oxidative stress, increasing the mitochondrial membrane potential, inhibiting cell apoptosis and promoting mitochondrial biogenesis in rats with Parkinson’s disease [22]. In agreement with previous studies, we also found signify that the NRP-1-conferred protection against cerebral I/R injury is modulated by the Wnt/β-catenin signaling pathway. Our results show that the Wnt/β-catenin signaling pathway may serve as a promising therapeutic candidate for neuroprotection.

Furthermore, the results of MD simulation and molecular docking illustrated a tight and stable integration of NRP-1 with Wnt1 and that of β-catenin with GSK-3β, Axin, and APC. Besides, the calculation of free energy revealed that the binding affinity of NRP-1 and β-catenin relies on H-bond and hydrophobic interaction, confirming the reliability of the docking model. Consequently, NRP-1 promotes the depolymerization of APC, Axin, and GSK-3β multimers through activating Wnt1 and increases the cellular deposition of β-catenin.

In conclusion, our study demonstrates that the overexpression of NRP-1 ameliorates neurological function in cerebral I/R-injured rats by promoting mitochondrial structural repair and functional recovery, in which NRP-1-conferred neuroprotection is mediated by the Wnt/β-catenin signaling pathway. These findings signify that NRP-1 may act as a promising candidate in treating cerebral I/R injury.

## Data Availability

The datasets used and/or analysed during the current study are available from the corresponding author on reasonable request.
